# Tailoring Defects in B, N-Codoped Carbon Nanowalls for Direct Electrochemical Oxidation of Glyphosate and its Metabolites

**DOI:** 10.1021/acsami.4c04478

**Published:** 2024-07-05

**Authors:** Mattia Pierpaoli, Paweł Jakóbczyk, Mateusz Ficek, Bartłomiej Dec, Jacek Ryl, Bogdan Rutkowski, Aneta Lewkowicz, Robert Bogdanowicz

**Affiliations:** † Faculty of Electronics, Telecommunications and Informatics, Gdańsk University of Technology, 11/12 Gabriela Narutowicza Street, Gdańsk 80-233, Poland; ‡ Institute of Nanotechnology and Materials Engineering, Gdańsk University of Technology, 11/12 Gabriela Narutowicza Street, Gdańsk 80-233, Poland; § Faculty of Metals Engineering and Industrial Computer Science, AGH University of Krakow, A. Mickiewicza 30, Krakow 30-059, Poland; ∥ Faculty of Mathematics, Physics and Informatics, University of Gdańsk, Wita Stwosza 57, Gdańsk 80-308, Poland

**Keywords:** vertical graphene, defect engineering, heterodoping, electrochemical oxidation, glyphosate, density functional theory (DFT)

## Abstract

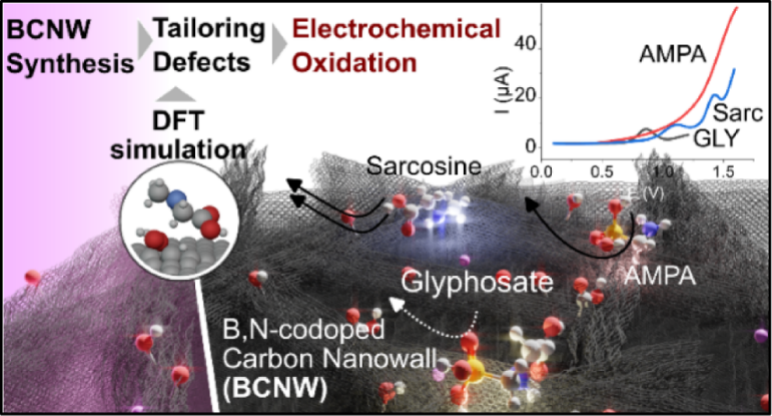

Tailoring the defects in graphene and its related carbon allotropes has great potential to exploit their enhanced electrochemical properties for energy applications, environmental remediation, and sensing. Vertical graphene, also known as carbon nanowalls (CNWs), exhibits a large surface area, enhanced charge transfer capability, and high defect density, making it suitable for a wide range of emerging applications. However, precise control and tuning of the defect size, position, and density remain challenging; moreover, due to their characteristic labyrinthine morphology, conventional characterization techniques and widely accepted quality indicators fail or need to be reformulated. This study primarily focuses on examining the impact of boron heterodoping and argon plasma treatment on CNW structures, uncovering complex interplays between specific defect-induced three-dimensional nanostructures and electrochemical performance. Moreover, the study introduces the use of defect-rich CNWs as a label-free electrode for directly oxidizing glyphosate (GLY), a common herbicide, and its metabolites (sarcosine and aminomethylphosphonic acid) for the first time. Crucially, we discovered that the presence of specific boron bonds (BC and BN), coupled with the absence of Lewis-base functional groups such as pyridinic-N, is essential for the oxidation of these analytes. Notably, the D+D* second-order combinational Raman modes at ≈2570 cm^–1^ emerged as a reliable indicator of the analytes’ affinity. Contrary to expectations, the electrochemically active surface area and the presence of oxygen-containing functional groups played a secondary role. Argon-plasma post-treatment was found to adversely affect both the morphology and surface chemistry of CNWs, leading to an increase in sp^3^-hybridized carbon, the introduction of oxygen, and alterations in the types of nitrogen functional groups. Simulations support that certain defects are functional for GLY rather than AMPA. Sarcosine oxidation is the least affected by defect type.

## Introduction

Defect engineering has emerged as a key approach to the manipulation of engineered nanomaterials because it allows for the precise tailoring and enhancement of material properties, leading to improved performance, novel functionalities, and a better understanding of fundamental mechanisms. Carbon nanowalls (CNWs), also known as carbon nanosheets, graphene walls, vertical graphene, graphene nanoflakes, and graphene nanopetals, represent a class of sheet-like nanostructures characterized by sharp edges, consisting of vertically self-standing few-layer graphene sheets. The predominance of edge defects, together with the presence of functional groups, topological defects, heteroatom substitutions, and vacancies, makes CNWs a rich and attractive electrode for electrocatalytic and electrochemical applications.^1^ These defects play a crucial role in improving the catalytic efficiency due to the higher density of active sites and fine-tuning of the local electronic structure, which in turn facilitates the rate-determining step of the reaction.^2^ Indeed, different types of defects can influence the interaction between the carbon transducer and target molecules, allowing the selective oxidation of specific analytes, such as sp^3^/sp^2^ carbon ratio,^3^ or boron-doping in diamond.^4^ By incorporating specific defect types or functional groups, the response of the sensor can be tuned to interact preferentially with target molecules while minimizing interference from other species, and defects can act as anchoring sites for functional molecules or nanoparticles, enabling the attachment of specific recognition elements or increasing the surface area for analyte adsorption. Nitrogen defects and boron dopants have been shown to effectively promote light trapping, charge separation, and valence band downshift.^5^ The literature indicates that when electron-rich nitrogen is used for doping, the carbon atoms in proximity to the nitrogen become catalytic centers for the oxygen reduction reaction (ORR). This is attributed to the activation of the π electrons through conjugation with the nitrogen’s lone pair of electrons. Conversely, in the case of boron doping, which introduces an electron deficiency, the boron sites themselves turn into active points for ORR. Here, the activation occurs as the π electrons are conjugated with the empty 2p_*z*_ orbital of the boron atoms.^6^ The Ar cluster ion beam is a versatile tool for graphene cleaning, defect engineering, and nanoporous or edge-rich graphene fabrication.^7^ Kim and colleagues investigated the interaction between Ar cluster ions and graphene (both supported and suspended) and found that the ion beam can form nanopores in suspended graphene while maintaining the lattice structure of the basal plane.^8^ Furthermore, edge carbon atoms are more active for the ORR than those in the basal plane, and this activity may arise from the delocalized charge distribution on the edge carbon atoms.^9^ Indeed, the vacancy defect induced by introducing nitrogen functionalities in CNW can result in enhanced catalytic activity by reducing the energy barrier of rate-limiting reactions.^10^ Nevertheless, both experimental and theoretical studies have shown that the presence of structural defects (edges, vacancies, voids, boundaries, etc.) plays a crucial role in determining the catalyst activity since the electrochemical reactions are most likely to occur at the defective sites.^9,11^

Rapid detection of glyphosate (GLY) can be achieved indirectly by aptamer-based sensors with a limit of detection (LOD) of 0.3 μM^12^ or by a variety of molecularly imprinted polymers that have been developed;^13^ however, direct detection is challenging. In their zwitterionic form, the chemical groups within the GLY molecule are unable to undergo reduction or oxidation at low potentials. In 1976, Bro̷nstad and Friestad^14^ defined “GLY itself to be polarographically inactive, whereas its N-nitroso derivative, to which it is readily converted in aqueous solutions, gave a good response.” The working electrode used was a dropping mercury electrode, and a peak was found at a potential of −0.78 V (vs SCE). Indeed, mercury could withstand a wide potential window, but it suffers from many problems related to its application in real-world settings due to poisoning and environmental pollution. GLY has been detected in water using copper-based compounds,^15^ electrogenerated copper ions,^16^ and copper-containing compounds within a multiwall nanotube matrix.^17^ In fact, in alkaline media, GLY functions as a tridentate ligand in the GLY/Cu complex via amine nitrogen, carboxyl, and phosphonate oxygens.^18,19^ Direct electrochemical detection of GLY on a carbon paste electrode was performed by Oliveira et al.^20^ who observed a nonreversible oxidation peak at +0.95 V vs Ag/AgCl; similarly, Caceres-Jensen and colleagues observed that a single and broad oxidation peak was obtained on MWCNT/GCE at a potential close to +0.05 V vs SCE^21^ (35 mV vs Ag/AgCl 3 M KCl). In our previous work, we demonstrated the exceptional versatility of boron/nitrogen-codoped carbon nanowalls in electrochemical sensing, both for direct detection^22^ and as functional substrates for tailoring supramolecular assemblies,^23^ also enhancing the electrochemical binding of perfluorooctane sulfonate (PFOS) in molecularly imprinted polymers.^24^ Carbon nanowalls, unlike planar carbon allotropes, facilitate fast electron transfer due to their high surface area and dense edge population, which are optimal sites for functional group attachment. This structure also influences their electronic and electrochemical properties, distinguishing them from bulk materials. Additionally, an increased surface area enhances the electrochemically active surface area (EASA), boosting oxidant electro-generation. Samples with the highest specific surface areas and large *I*_D_/*I*_G_ ratio, indicating numerous defects at corners, edges, and holes, exhibit improved oxidation activity, suggesting that larger areas rich in such structural defects enhance oxidation performance.^25^ However, how the various defects resulting from both process synthesis and postmodification affect the morphology and what role the surface chemistry of the carbon plays in the electrochemical response are still open questions.

In this study, B, N-codoped carbon nanowall (BCNW) electrodes were synthesized and further tailored to achieve various defect types (i.e., vacancies, edges). The electrodes were fabricated through microwave plasma-enhanced chemical vapor deposition, utilizing the simultaneous incorporation of nitrogen and boron dopants during growth to form codoped BCNW structures. Tailoring defects in BCNW was carried out by varying the gas precursor composition and applying Ar plasma post-treatment, thus allowing for the precise customization of the defect types and functional groups in the vertical graphene stacks. After thorough characterization, we concentrated our efforts on enhancing the electrochemical oxidation of GLY and its key byproducts, aminomethylphosphonic acid (AMPA) and sarcosine (Sarc). These substances were specifically selected for the study due to their widespread presence in the environment and their particular reactivity toward direct electrochemical oxidation techniques. Additionally, MD (molecular dynamics) simulations were utilized to support the experimental results and highlight how the different types of defects in BCNW affect the oxidation of GLY, AMPA, and Sarc.

## Results and Discussion

### Synthesis and Characterization of BCNW

**Figure 1:**
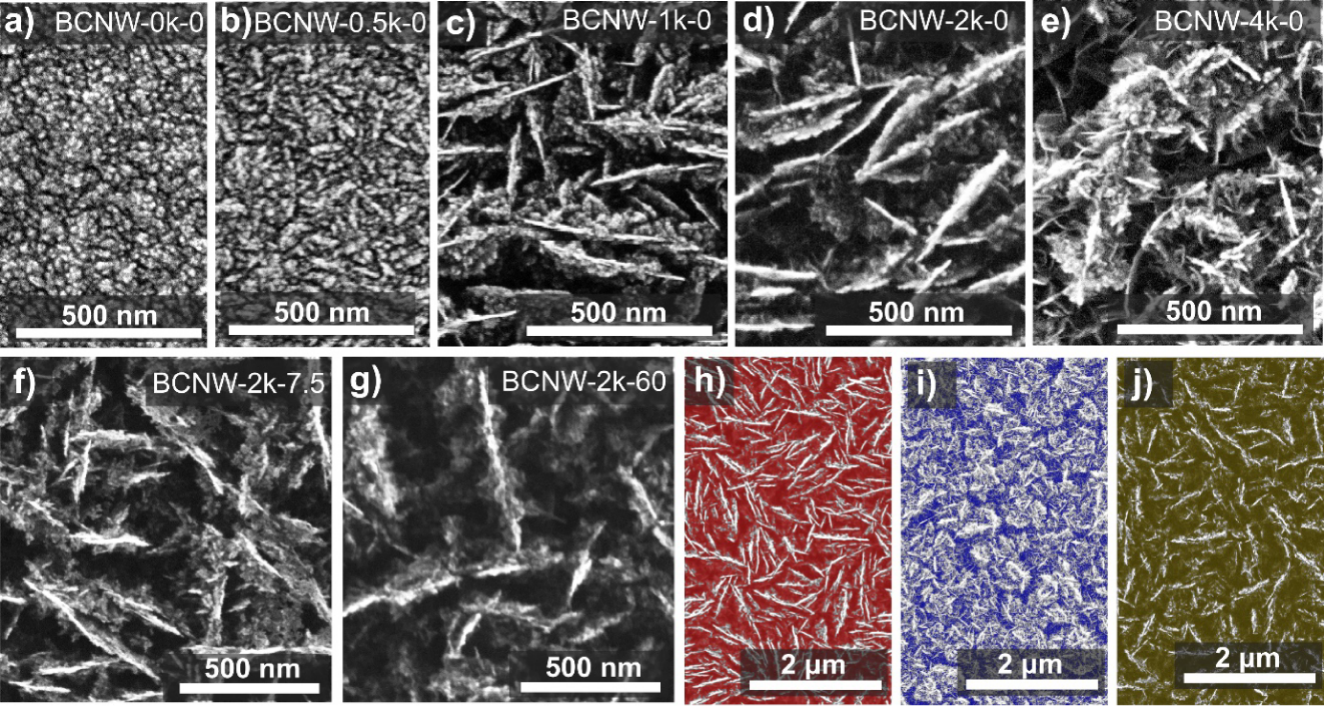
SEM images of the BCNW samples before (a–e) different boron concentrations and (f–i) BCNW-2k after Ar-plasma treatment at different durations.

From the SEM micrographs shown in Figure 1a–e, it is evident that boron plays a predominant role in the synthesis of BCNW.^26^ As the boron concentration in the gas mixture increases, the formation of characteristic lamellar walls begins for [B]/[C] > 500, with the largest developed surface area observed in the BCNW-2k sample (Figure S1 and Table S1). The presence of diborane catalyzes the formation of well-defined graphitic nanostructures and secondary walls perpendicular to the main ones. For the [B]/[C] = 4000 sample, an excess growth is observed (Figure 1e).

Conversely, post-treatment under Ar plasma etches the secondary walls even after a short treatment duration of 7.5 min (Figure 1f). Prolonged treatment does not significantly alter the morphology compared with shorter treatments (Figures 1g, S2).

SEM analysis reveals that the space within the walls constitutes approximately 65% of the total projected area for BCNW-2k-0 (Figure 1h). This percentage decreases with increasing boron doping because the activity of boron leads to the renucleation of secondary walls and the formation of “closed” porosity, ultimately halving the total projected area (Figure 1i). Conversely, Ar-plasma treatment increases this space to 80% of the total projected area due to preferential etching of the secondary perpendicular walls (Figure 1j).

**Figure 2:**
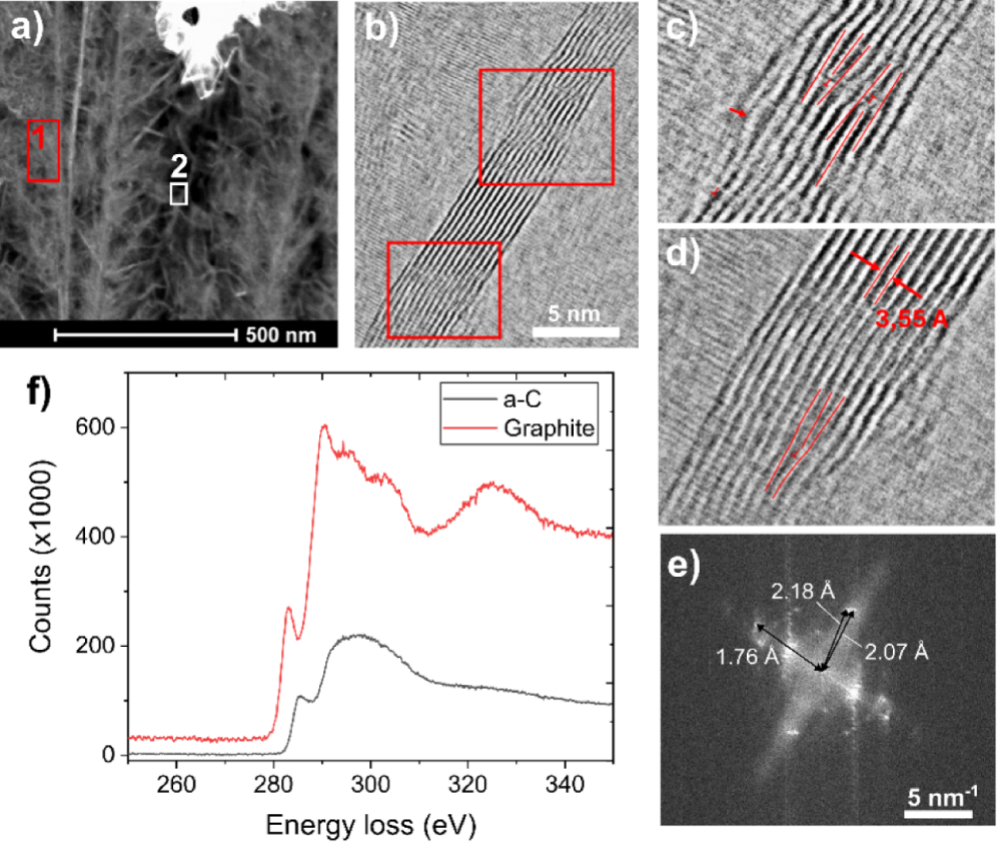
(a–d) STEM images of the BCNW samples; (e) FFT of part b; (f) EELS spectrum obtained from areas marked in part a as “1”(red) and “2”(black).

STEM-HAADF analyses were performed to thoroughly examine the microstructure of BCNW (Figures 2a and S3). Two distinct regions were identified: a lighter gray contrast region (labeled as area “1”) and a darker region (labeled as area “2”). Core loss spectra ranging from 250 to 350 eV were obtained from these areas (as shown by squares in Figure 2a). EELS studies (Figure 2f) indicated the presence of graphitic carbon in region “1,” whereas region “2” was found to consist of amorphous carbon. Both spectra corroborate the findings reported in literature.^27,28^

The development of BCNW was further analyzed to elucidate the structure of the nanowalls. Two distinct types of BCNW nanostructures were identified and visualized. The first type is characterized by long fibers that originate near the substrate surface and grow perpendicular to it, spanning nearly the full depth of layer.^29^ Complementing these are shorter, randomly oriented BCNWs. Additionally, an amorphous carbon phase is present, situated between the graphitic phases. In Figure 2b (STEM-BF), the microstructure of a typical BCNW is shown. Red rectangles indicate the areas shown at higher magnification in Figure 2c,d, revealing the presence of linear defects (dislocations). Red lines were drawn for guidance and the reader’s comfort. Additionally, a red arrow in Figure 2c indicates distortions in the single-layer growth, where the distance between layers is greater than the average. Figure 2e shows the fast Fourier transform (FFT) of Figure 2b. Indices’ characteristics of planes with interplanar distances of 2.18, 2.07, and 1.76 Å were found, which, considering measurement error, can be assigned to the (0,0,4), (1,0,1), and (1,0,3) planes of graphite, respectively. This is in high agreement with the EELS results (Figure 2f).

**Figure 3:**
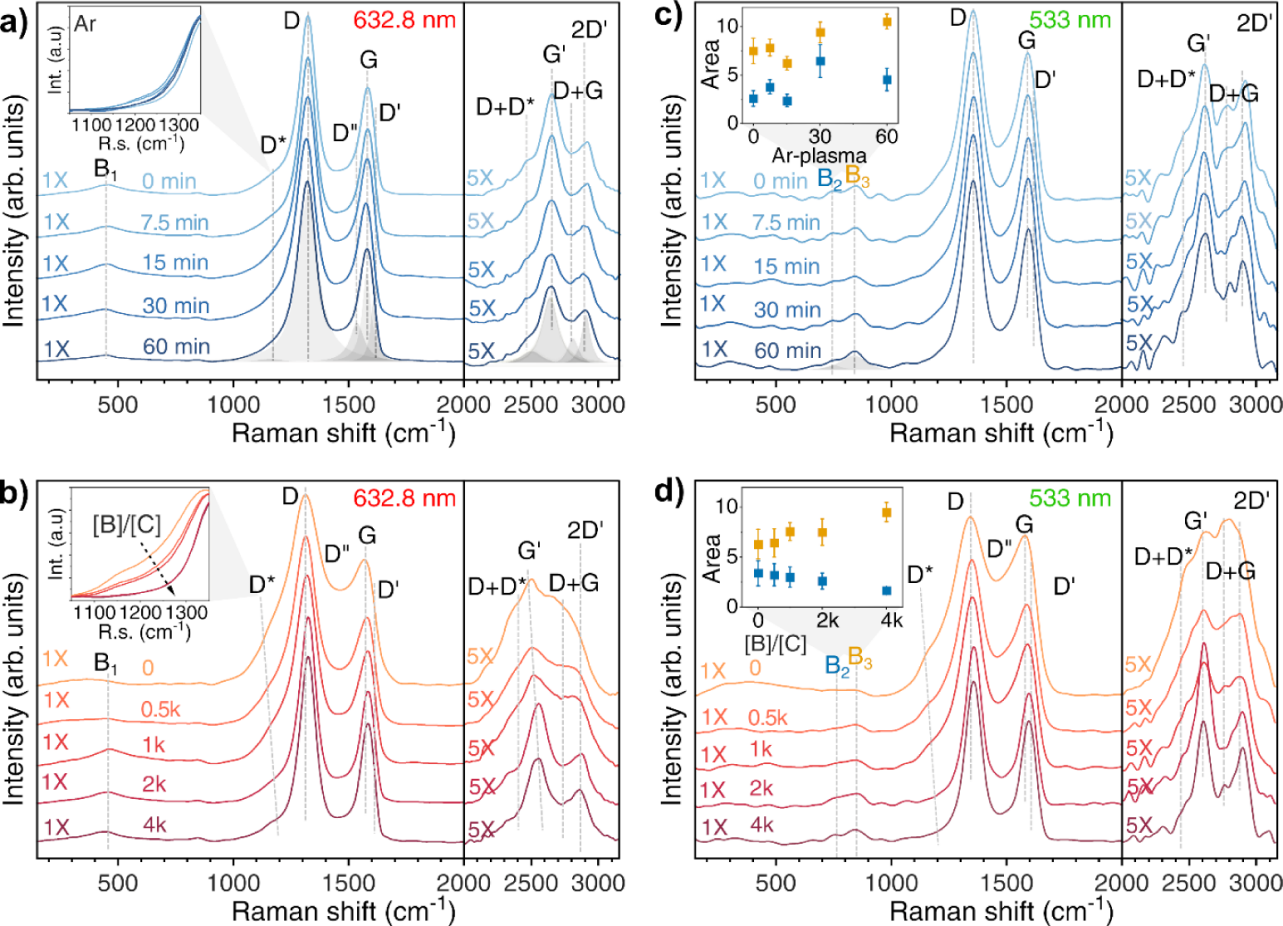
Raman spectra for the BCNW collected using different excitation lasers for different (a,c) Ar-plasma treatment and (b,d) [B]/[C] ratios.

Raman spectra collected from different excitation lasers are shown in Figure 3. A deconvolution using 5 peaks for the 900–1700 cm^–1^ interval was chosen based on our previous studies and the literature.^30^ The G-band peak is the first-order Raman band of the fully sp^2^-hybridized carbon, while the D-band is a defect-activated band in the sp^2^-hybridized carbon, indicating imperfections in the graphene lattice. In addition to the G peak, an additional shoulder peak, known as the D’ peak, indicates defects caused primarily by localized vibrational modes of randomly distributed impurities at the edges of the graphitic material.

With an increasing [B]/[C] ratio, the D peak shifted to higher wavenumbers from 1342 to 1360 cm^–1^ when excited by a 532 nm laser or from 1313 to 1325 cm^–1^ when excited by a 633 nm laser. Similarly, the G peak showed a shift to higher wavenumbers with the increasing [B]/[C] ratio, reaching 1598 cm^–1^ (532 nm laser) and 1586 cm^–1^ (633 nm laser). In contrast, the intensity of the D″ peak decreased with the increasing [B]/[C] ratio. The origin of the D* peak has been suggested to be due to the presence of *trans*-polyacetylene in the grain boundaries,^31,32^ observed with the increasing CH_4_/H_2_ ratio at a nanometer BDD grain size,^33^ vibrations of carbon atoms restricted by oxygen-containing groups^34^ or the high density of edges.^35^ In our case, we observed the D* intensity decreasing with more boron being incorporated into the BCNW surface,^36^ thus resulting in a decrease of the C–H bond in favor of B–C and B–H bonds, while its position remained constant at around 1160 cm^–1^ for both excitation sources (Figure 3a,b insets). The D” peak can be attributed to the presence of interstitial or out-of-plane defects and *trans*-polyethylene,^30^ which is one of the two possible edges of graphene sheets.^35^ BCNWs exhibit bands in higher shift regions (>2000 cm^–1^), namely, the G′ (2D), D+G, 2D’, and D+D* overtone modes. The presence of the D band and a weak 2D peak is characteristic of multilayered graphene with numerous structural defects. Pyrrolic (Pyr) and pyridinic (Pyd) nitrogens are expected to be less readily detected by Raman scattering. Indeed, they are responsible for the emergence of multiple new peaks within the 1500 to 1600 cm^–1^ range. These peaks tend to merge with the G band, complicating their identification. Moreover, the Pyr, Pyd, and chemisorbed nitrogen systems collectively display a peak at approximately 1610 cm^–1^,^37^ which may be confused with the D’ band. The boron-to-carbon ratio in the carbon material showed several effects. In the overtone region, the G′ peak remained constant at 533 nm excitation but shifted from 2613 to 2657 cm^–1^ with the increasing [B]/[C] ratio when excited by a 633 nm laser. In addition, the position of the 2D’ peak shifted to higher wavenumbers, from 2920 to 2948 cm^–1^ with the increasing [B]/[C] ratio. Most interestingly, the intensity of the D+D* peak became much more distinguishable than the D* in the fundamental region and showed a decreasing trend with the increasing boron concentration.^38,39^ The relatively wide peak (B_1_) at 500 cm^–1^ is typical of heavily doped boron-doped diamond, and it is easily distinguishable with a 633 nm laser excitation. The weak band at 850 cm^–1^ can be attributed to H bonded to sp^2^-C.^40^ It is more intense for the undoped and lowly doped CNWs, and it increases with increasing Ar treatment. At 750 cm^–1^, a small band (B_2_) arose in the range of the phonon density of states of graphite.^41^ C–H stretching modes can be seen for UV excitation, due to the overwhelming contribution of sp^2^-C under visible excitation. However, a laser wavelength λ*L* = 514.5 nm has also been found to be effective for the purpose.^42^ Indeed, the weak band only appeared under 533 nm excitation (B_3_). It is interesting to see that B_3_ increased with both the increasing boron doping and Ar-treatment duration. The A(D)/A(G) ratio slightly increased (λ_ex_ = 632.8 nm) with the increasing Ar-plasma treatment duration; a similar behavior was described by Kim et al. for pristine graphene samples irradiated with increasing Ar-ion doses^8^ to indicate the increased defect density, while it is almost constant for λ_ex_ = 533 nm (Figure S4e). On the other hand, the A(D)/A(G) decreased with the increasing boron incorporation (Figure S4b), together with the FWHM(G) (Figures S4a, S4d), suggesting a decrease in the level of disorder, as also supported by the longer wall dimensions.^26,43^

**Figure 4:**
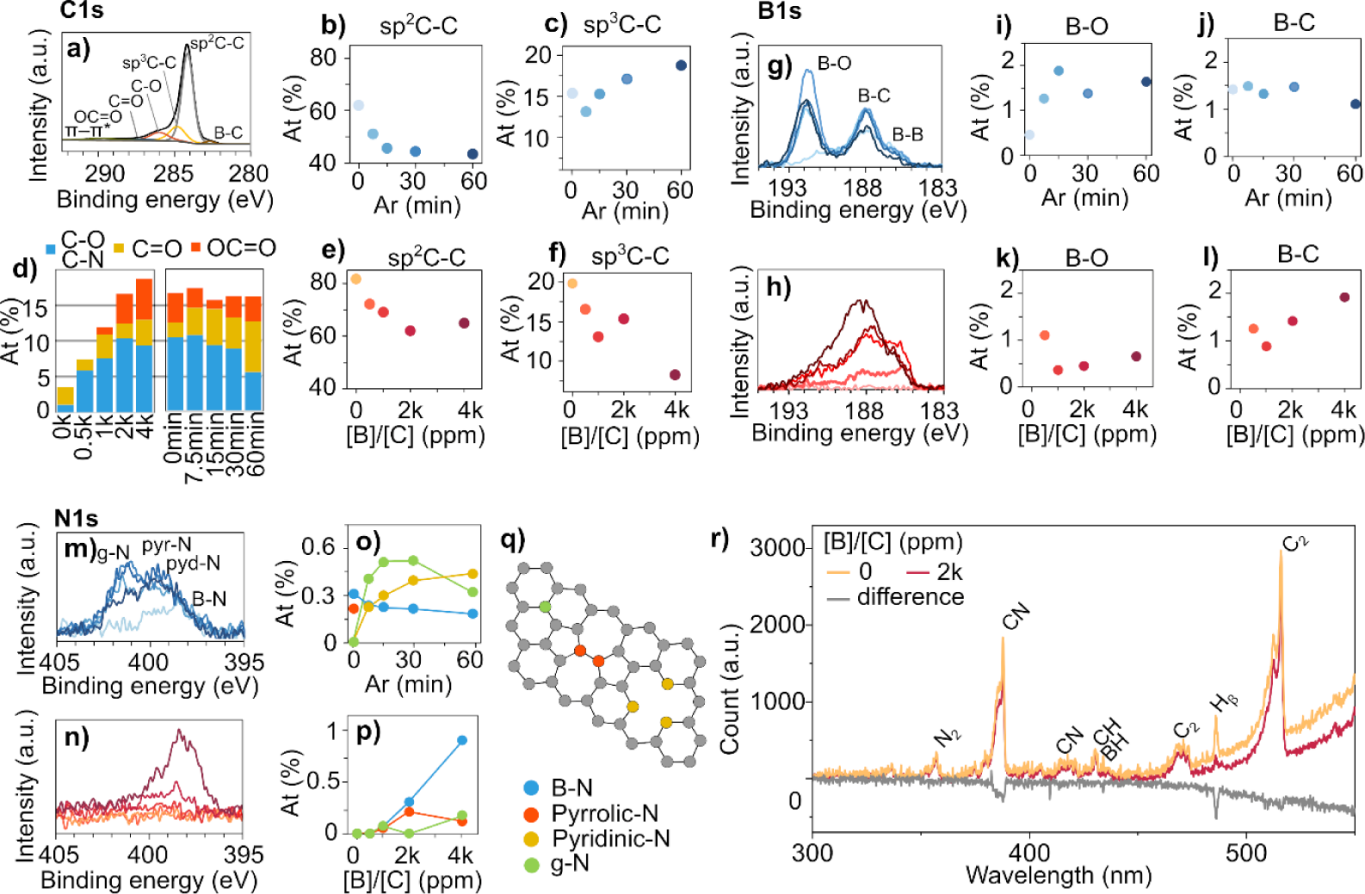
(a) High-resolution XPS spectra of the carbon region (C 1s) for the BCNW2k-0. Effect of the Ar-plasma treatment for the (b) sp^2^-C and (c) sp^3^-C, as well as (e,f) for the different [B]/[C] and (d) other bonds. (g,h) High-resolution XPS spectra of the B 1s region. Estimation of the (i,k) B–O and (j,l) B–C contents as a function of the Ar-treatment duration and [B]/[C]. (m,n) High-resolution XPS spectra of the N 1s region. (o–q) Quantification of the various bonds as a function of the Ar-treatment duration and [B]/[C]. (r) Optical emission spectra of microwave plasma recorded during the PECVD growth of BCNW in the presence and absence of B_2_H_6_.

The XPS study of B-CNW samples obtained by Ar plasma treatment with different boron doping concentrations revealed chemical changes in the surface of the samples. The C 1s core level of the BCNW, plotted in Figure 4a, shows a predominant peak related to the presence of the C sp^2^ hybrid bond, slightly asymmetric with a tail toward high binding energy.

The most intense peak was the sp^2^ component at 284.4 eV, whose area decreased from 62% to 44% with increasing Ar treatment time (Figure 4b), followed by peaks at 285.1 eV corresponding to sp^3^-C (Figure 4c). The decrease in the sp^2^-C and increase in the sp^3^-C concentration is consistent with other studies^1,44^ and was due to the high energy ions and radicals in the argon plasma, which can disrupt the π network of graphene, breaking some of the C=C bonds and introducing sp^3^ hybridized carbon atoms, making their ratio a useful indicator of the level of defects present. At the same time, a clear increase in the intensity of the O 1s peak (Figures S5 and S6a) was observed compared to the untreated samples, although the cumulative presence of C–O, C=O, and OC=O showed small changes within the plasma-treated samples. Specifically, the increase in intensity at 286.3 eV with the increasing boron concentration may be due to the increase in C–N rather than C–O, since the two groups overlap,^45^ and no significant changes were observed in the O 1s region (Figure S6b). Similarly, the increase in the carboxylic group could be attributed to π–π* transitions instead. Indeed, the presence of nitrogen in diborane is known to enhance BCNW growth.^26^ Interestingly, the sp^2^-C decreased with the increasing boron doping (Figure 4e), as well as the sp^3^-C (Figure 4f). For the B 1s core, the XPS profiles of the BCNW samples showed three deconvoluted peaks of B–O (∼191.1 eV), B–C (∼188.1 eV), and B–B (186.1 eV) bonds^1,39^ The intensity of the B 1s peak increased with the increasing B/C flow ratio. While a quasilinear correlation with the presence of boron and the [B]/[C] ratio was observed (Figure 4j), the B–O bond intensity increased with plasma treatment. The N 1s core level was deconvoluted into four widely accepted distinct peaks corresponding to N–B (∼398.4 eV), Pyd N (∼399.6 eV), Pyr N (∼400.2 eV), and graphitic N (∼401.5 eV) bonds^46^ (Figure 4m), although the core-level binding energies of graphitic and Pyr (N 1s) can be difficult to distinguish.^47^ On the other hand, the N–B assignment is unambiguous and is noticeable for [B]/[C] > 1k ppm (Figure 4n). On the other hand, the plasma treatment introduced graphitic N (Figure 4o). Figure 4p shows the presence of only N–B, Pyd, and Pyr N for the BCNW at higher B doping levels (2k and 4k), while graphitic N is absent. Interestingly, the Pyr N increased with the [B]/[C] ratio; indeed, at the same N_2_, CH_4_, and H_2_ content, the introduction of B_2_H_6_ modified the plasma composition, among which the presence of CN and CH radicals (Figure 4r).^26^

### BCNW Electrochemical Behavior

The peak-to-peak separations (Δ*E*_p_) are 652, 402, 127, and 138 mV for the BCNW-0.5k-0, BCNW-1k-0, BCNW-2k-0, and BCNW-4k-0 electrodes, respectively. The lower peak-to-peak separation for the BCNW-2k-0 electrode indicates faster electron transfer kinetics compared to the BCNW-0.5k-0, BCNW-1k-0, and BCNW-4k-0 (Figure S7). This is due to the easier adsorption of electroactive ions by the sp^2^-C and its effect on the rate of the redox reaction.^3^ EIS can provide a more detailed study of the processes occurring at the electrode/electrolyte interfaces. The analysis focused on the evaluation of the electrode charge transfer resistances (*R*_ct_). The value of the solution resistance (*R*_s_) is not only influenced by the type of electrolyte but also by the active surface area of the electrode. The smaller active surface area, together with the lower the value of *R*_s_, suggests that the lowest Δ*E*_p_ of BCNW-2k-0 may arise from the intrinsic atomic structure and specific boron-doping, rather than indirectly from its morphology (Table 1). In the case of electrodes treated with argon plasma after growth, the lowest value is for the BCNW-2k-30.

**Table 1:** Electrochemical Characteristics Obtained from Cyclic Voltammetry (CV)—Including Δ*E*_p_ at a Scan Rate of 20 mV s^–1^—and Electrochemical Impedance Spectroscopy (EIS)—Featuring *R*_ct_ and *R*_s_ at Open Circuit Voltage—for BCNW Electrodes with Different Boron Doping^a^

Element	0k	0.5k	1k	2k	4k
Δ*E*_p_ (mV) @ 20 mV s^–1^	–	652	402	127	138
*R*_s_ (kΩ cm^–2^)	1.59 (0.073)	1.54 (0.016)	1.44 (0.022)	1.17 (0.002)	1.38 (0.02)
*R*_ct_ (kΩ cm^–2^)	749 (4.5)	16.5 (0.22)	38.6 (0.72)	0.283 (0.005)	3.52 (0.096)
*Q*_0_ (μF cm^–2^ s^–1^)	0.58 × 10^–6^ (7.6 × 10^–9^)	8.69 × 10^–6^ (6.3 × 10^–8^)	2.73 × 10^–6^ (3.4 × 10^–9^)	17.8 × 10^–6^ (1.1 × 10^–6^)	9.60 × 10^–6^ (0.38 × 10^–6^)
α (-)	0.96 (0.005)	0.70 (0.007)	0.91 (0.005)	0.87 (0.01)	0.90 (0.01)
*C*_eff_ (F cm^–2^)	0.53 × 10^–6^ (3.8 × 10^–8^)	3.78 × 10^–6^ (1.5 × 10^–8^)	2.17 × 10^–6^ (2.7 × 10^–9^)	8.24 × 10^–6^ (5.9 × 10^–9^)	6.68 × 10^–6^ (4.1 × 10^–8^)
*A*_w_ (kΩ cm^–2^ s–1/2)	31.5 (0.82)	1.87 (0.19)	8.65 (0.64)	1.64 (0.013)	4.88 (0.15)
EASA (cm^–2^)	–	1.76 × 10^–2^ (8.6 × 10^–3^)	3.46 × 10^–2^ (3.8 × 10^–4^)	1.19 × 10^–2^ (2.6 × 10^–3^)	7.36 × 10^–2^ (8.6 × 10^–4^)

a These measurements were conducted in 0.05 M PBS containing 2.5 mM K_3_[Fe(CN)_6_]/K_4_[Fe(CN)_6_] in a 1:1 weight ratio using Ag/AgCl wire as the reference electrode. The standard error is reported in brackets.

The charge-transfer resistance, representing the resistance of the electrons transferred through the electrode/electrolyte interface, includes both faradaic and nonfaradaic components. The faradaic components are generated by electron transfer across the interface with solution resistance, and the nonfaradaic component is caused by the electric double layer. When charge transfer occurs at the interface, mass transfer of the reactants and products plays a role in determining the electron-transfer rate, which is dependent on the consumption of the oxidant and the production of the reducing agent near the electrode surface.^48^ The resistance *R*_ct_ represents the charge-transfer resistance and is influenced by the electrode porosity^49^ and chemical groups at the electrode surface.^50,51^ The sample with the lowest *R*_ct_ value among the various boron-doped samples (as listed in Table 1) was found to be BCNW-2k-0. This can be attributed to its enhanced adsorption capabilities, primarily driven by the presence of hydroxyl, carbonyl, and carboxyl groups (Figure 4d). Similarly, the BCNW-4k-0 contains a relevant amount of hydroxyl, carbonyl, and carboxyl groups as well, and it has a larger active surface area, as indicated in Table 1 (EASA).

For the electrodes after different argon plasma treatment times, the proportion of oxygen-containing functional groups is comparable; however, the *R*_ct_ differences are significant. The lowest *R*_ct_ is for the sample after 30 min of plasma treatment, and it also has the highest capacity, which indicates an increased surface area, and an alpha coefficient of one (Table 2), which indicates a low effect of diffusion on the transport of electroactive species.

**Table 2:** Electrochemical Characteristics Obtained from Cyclic Voltammetry (CV)—Including Δ*E*_p_ at a Scan Rate of 20 mV s^–1^—and Electrochemical Impedance Spectroscopy (EIS)—Featuring *R*_ct_ and *R*_s_ at Open Circuit Voltage—for BCNW Electrodes with Different Treatment Times^a^

Element	0 min	7.5 min	15 min	30 min	60 min
Δ*E*_p_ (mV) @ 20 mV s^–1^	127	97	100	74	92
*R*_s_ (kΩ cm^–2^)	1.17 (0.002)	1.26 (0.013)	1.19 (0.001)	0.627 (0.001)	1.00 (0.002)
*R*_ct_ (kΩ cm^–2^)	0.283 (0.005)	0.648 (0.013)	0.232 (0.007)	0.087 (0.004)	0.399 (0.005)
*Q*_0_ (μF cm^–2^ s^–1^)	17.8 × 10^–6^ (1.1 × 10^–6^)	7.93 × 10^–6^ (4.6 × 10^–7^)	7.57 × 10^–6^ (4.6 × 10^–7^)	13.1 × 10^–6^ (4.4 × 10^–7^)	14.0 × 10^–6^ (6.2 × 10^–7^)
α (-)	0.87 (0.01)	0.90 (0.01)	0.96 (0.008)	1.00 (0.001)	0.89 (0.008)
*C*_eff_ (F cm^–2^)	8.24 × 10^–6^ (5.9 × 10^–9^)	4.32 × 10^–6^ (6.6 × 10^–9^)	5.72 × 10^–6^ (3.5 × 10^–9^)	13.1 × 10^–6^ (1.8 × 10^–9^)	7.21 × 10^–6^ (3.3 × 10^–9^)
s*A*_w_ (kΩ cm^–2^ –1/2)	1.64 (0.013)	2.42 (0.012)	1.86 (0.027)	2.72 (0.028)	2.34 (0.011)
EASA (cm^–2^)	1.19 × 10^–2^ (2.6 × 10^–3^)	6.91 × 10^–2^ (8.6 × 10^–4^)	9.16 × 10^–2^ (9.7 × 10^–4^)	8.88 × 10^–2^ (4.2 × 10^–4^)	6.70 × 10^–2^ (9.3 × 10^–4^)

a These measurements were conducted in 0.05 M PBS containing 2.5 mM K_3_[Fe(CN)_6_]/K_4_[Fe(CN)_6_] at a 1:1 weight ratio using an Ag/AgCl wire as the reference electrode.

The presence of more N-graphitic and sp^2^ C–N groups contributed to a lower *R*_ct_ and facilitated the adsorption of the electroactive species. Consequently, the BCNW-2k-30 sample achieved the lowest *R*_ct_, while the BCNW-2k-7.5 showed the lowest proportion of sp^2^ C–N groups and the highest *R*_ct_. Although the BCNW-2k-60 sample exhibited the highest number of sp^2^ C–N bonds, a higher *R*_ct_ of 399.1Ω was observed due to the lower amount of graphitic nitrogen compared to the 30 min argon plasma-treated sample.

The EIS spectra were analyzed and fitted with the EEC shown in Figure S8. Capacitance values (*C*_eff_), were estimated by EIS, from the CPE (Figure S9). The highest capacitance value for samples differing in boron doping was obtained for the BCNW-2k-0 sample, which can be related to the resulting p-type doping;^52^ moreover, the presence of graphitic and Pyd nitrogen in the electrode structure also enhances capacitance,^53^ contributing to the high capacitance observed in the BCNW-2k-30 electrode (Table 2).

In cyclic voltammetry, the linear relationships between the anode and cathode peak currents and the square root of the scan rate were characterized by the slopes presented in Table S2. A linear increase in the peak current with the square root of the scan rate indicates a consistent diffusion limitation for the redox processes. The slope, reflective of the kinetics of electrode reactions, exhibited variations with different levels of boron doping and plasma treatment durations. A higher absolute value of the slope indicates a more rapid electrode reaction. Initially, an increase in the slope was observed with higher levels of boron doping, reaching a peak for the 2k electrode, and then declining for the 4k electrode. However, data for the 0.5k electrode was excluded due to the irreversible nature of the electrode reactions, where Δ*E*_p_ exceeded 400 mV/s at a scan rate of 5 mV s^–1^. After the post-growth argon plasma treatment, a reduction in the slope was noted, reaching its lowest point after 60 min of treatment. The duration of plasma treatment influenced the presence of sp^2^-C, subsequently affecting the ease of adsorption and electrode reaction rate. A decrease in the amount of sp^2^-C was observed with longer argon plasma treatment times, while the amount of C–N initially decreased for the first 7.5 min of treatment and then showed an opposite trend. This variation in the presence of C–N sp^2^ bonds could explain the observed reduction in the slope for the BCNW 7.5 min sample compared to the BCNW 15 min, BCNW 30 min, and BCNW 60 min samples.^54,55^

### Quantum Calculations Results

**Figure 5:**
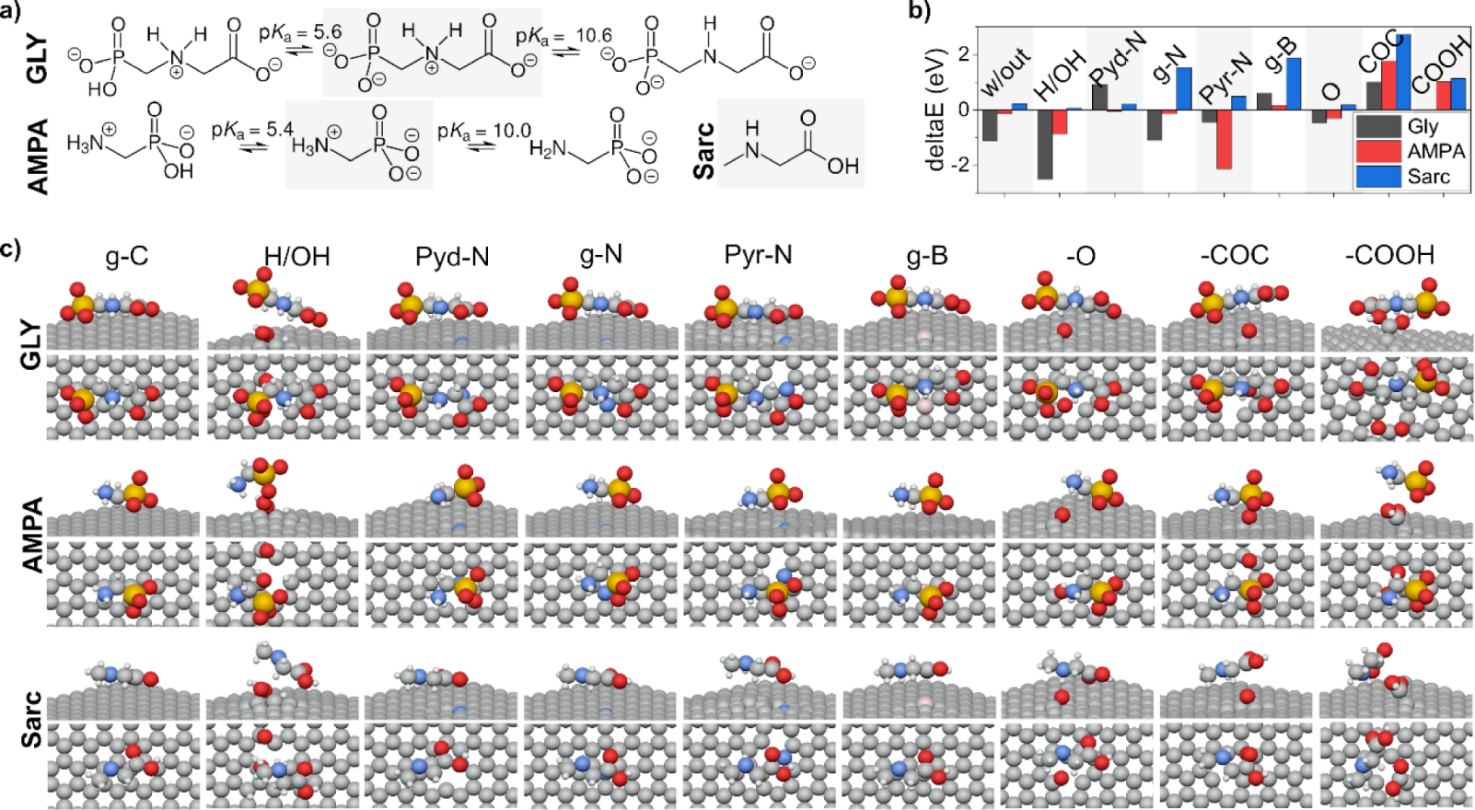
(a) Ionic states of GLY and AMPA. Highlighted in gray: the configuration employed for the simulation. (b) Adsorption energies Δ*E* of studied analytes at different BCNW defects. (c) Analyte orientation at BCNW defect sites (front and top view after optimization).

In the GLY electrochemical study, various factors influenced its activity, such as the pH, electrode material, and presence of different nitrogen configurations. At a pH of 7.4, deprotonated GLY formed due to the protonation of the nitrogen atom in the secondary amine and the deprotonation of the hydroxyl oxygen atom (Figure 5a).^17,56^

Molecular dynamics simulation results indicate that the type of defect on a surface can significantly influence the adsorption energy of a molecule. For GLY, the strongest adsorption was observed for the H-terminated/hydroxyl defect type with an energy of −2.51 eV, indicating a high interaction, while the weakest interaction was with the pyridinic-N defect with an energy of 0.93 eV, indicating either weak interaction or possibly even repulsion. The configuration without defects showed similar adsorption energy to the graphitic-N, suggesting analogous interactions. AMPA followed a behavior similar to GLY, a strong adsorption towards the H-terminated/hydroxyl defect type and pyrrolic-N. Unlike GLY, the absence of defects showed a weak interaction with AMPA (−0.13 eV). The adsorption energies for Sarc were generally lower than those of GLY and AMPA. Hydrogen termination showed the lowest adsorption energies, indicating the preferred interaction with Sarc.

### Unraveling Correlation Between BCNW Defect Type and Analytes Oxidation

**Figure 6:**
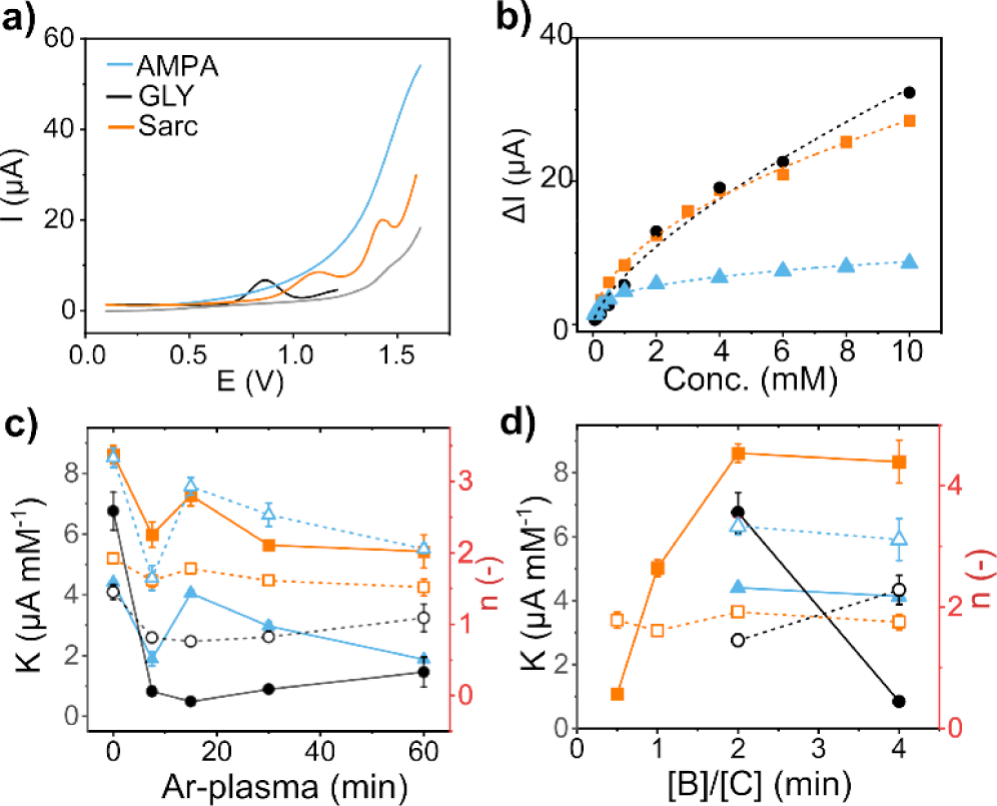
(a) DPV for GLY, AMPA, and Sarc and background in PBS. (b) Fit of the current peak to the analyte concentration using eq 2. Fitted *K* and *n* versus (c) the plasma treatment duration (d) and [B]/[C] ratio.

Indirect GLY electrochemical oxidation mechanisms are unlikely, as a phosphate buffer solution was used, limiting the production of potential oxidants, and a low potential was employed. The direct electrochemical oxidation of GLY involves the exchange of electrons between GLY and the working electrode’s surface, incorporating two protons and two electrons, resulting in the formation of cyanophosphonic acid and acetic acid.^20,57^ GLY, AMPA, and Sarc were found to be simultaneously oxidizable only on the BCNW-2k-0 (Figure 6a) and BCNW-4k-0. The correlation between the current difference in DPV and the concentration of specific analytes was fitted by using eq 2 (Figure 6b). The resulting values of *K* and *n* are reported against the plasma treatment duration (Figure 6c) and [B]/[C] ratio (Figure 6d).

**Figure 7:**
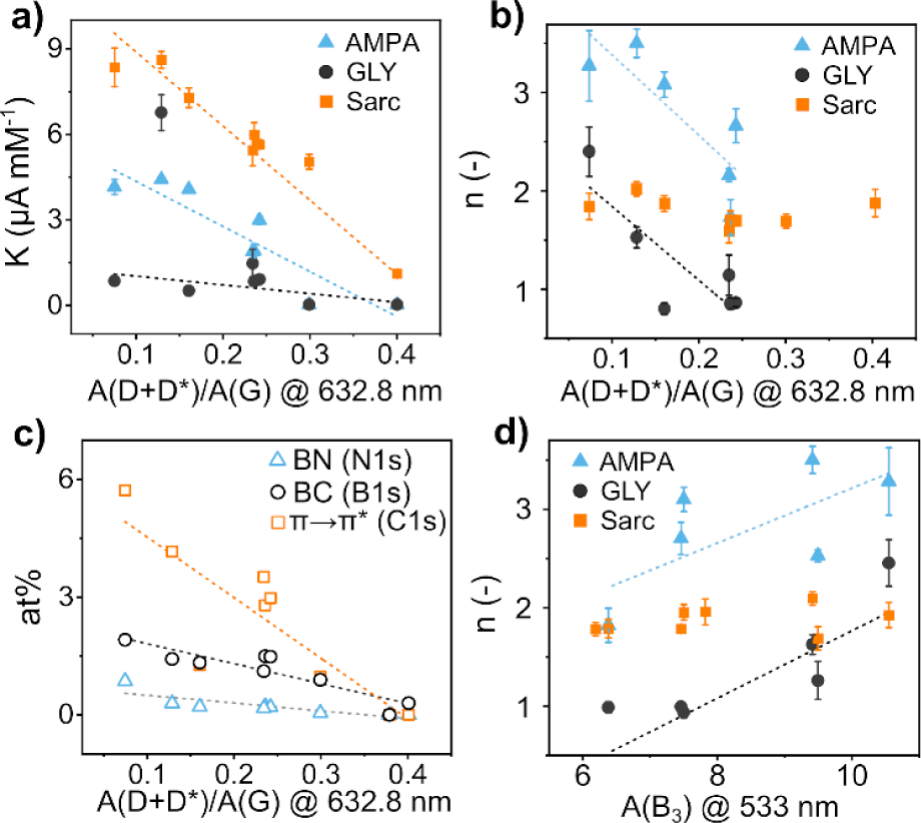
Correlation between the Raman D+D*/G area ratio and (a) *K*, (b) *n*, and (c) the XPS results. (d) Correlation between the B_3_ area and *n*.

Inverse correlations between *K, n* and the ratio of the D+D* over G peak areas were found for the three analytes (Figure 7a,b). Interestingly, only for Sarc, the favorability of the adsorption process seems to be independent from such indicator (Figure 7b). As discussed in section BCNW Synthesis and Modification, the specific second-order combinational Raman mode arose from the D* peak, the assignment of which is ambiguous in the literature. In particular, in our study, we found that the D+D* area decreased inversely with (1) the B–C, (2) the B–N bonds, and (3) the broad peak at the highest binding energy of C 1s, which has been attributed to the carboxylic or the π → π* shakeup satellite (Figure 7c).

Previous work by Santos et al.^57^ using a graphite oxide paste electrode in a BR buffer at pH 6 revealed the irreversible oxidation of the amine group of GLY at 1.31 V vs Ag/AgCl. They found that the kinetic parameters of the electrochemical reaction of GLY were significantly influenced by the presence of graphite oxide. The enhanced current measured was attributed to the oxygen-containing groups in the graphite oxide, which increased the catalytic activity of the electrode due to a higher chemical affinity between the protonated GLY nitrogen atoms and oxygen atoms. In our study of GLY, we did not find such a correlation; moreover, from the simulation results, all of the oxygenated defects possessed the highest values of Δ*E*, suggesting a repulsive behavior. Despite the noticeable correlation between π → π* shakeup satellite and the value of *n* (Figure 7c), the interpretation is not unambiguous, since the carboxylic group may partially overlap with the satellite peak (Figure 4a); however, it has been reported how the latter can be correlated with other process parameters (e.g., synthesis temperature),^58^ being related to the presence of aromatic structures. This aspect is also supported by the simulation results, in which the absence of defects is linked to favorable Δ*E* values for GLY and AMPA, but not for Sarc (Figures 5b and 6b). Another important factor is the absence of Pyd N (Figure 4o), which has a relatively high basicity according to the Lewis theory.^59,60^ However, as glyphosate began to deprotonate and carry a negative charge, it was likely to also act as a Lewis base, leading to a repulsive behavior and explaining the lack of electrochemical response in the Ar-treated samples. In contrast, the involvement of the lone pair of electrons of the nitrogen atom in the aromatic π-electron system of Pyr N made it less available for interaction with protons and exhibited a much lower basicity, thus facilitating effective GLY oxidation. The role of Pyr and Pyd N was also confirmed by the simulation, where they had negative and positive Δ*E* for GLY, respectively (Figure 5b). The net Lewis acidity or basicity of a B–N defect may depend on the overall structure since B and N could act as acid and base, respectively. It has been reported that graphitic (quaternary) nitrogen defects provide the least ability to donate electrons, meaning that their Lewis basicity is weaker than the others.^60^ Graphitic nitrogen is mostly found in plasma-treated samples, which may explain the lower affinity toward GLY and AMPA. Interestingly, we found that after testing the electrodes over multiple cycles of oxidation, followed by methanol washing and rinsing between cycles, they maintained consistent performance through 101 cycles. There was a slight shift to a higher potential in the oxidation peak (around 0.7 V) and a lower current after 101 cycles, but the overall signal and background remained stable (Figure S10).

GLY is rapidly biodegraded to its primary metabolite, AMPA, in which oxidation occurs at potentials above 1.5 V, suggesting a threshold voltage between 1.5 and 2.0 V (vs AgCl). This degradation process results in the formation of phosphate ions (PO_4_^3–^*)* and methylamine, facilitated by the C–P bond lyase.^61^ In our observations, a subtle shoulder of the oxidation peak appears to begin at about 1.5 V. At pH 7, AMPA is likely to be negatively charged due to the deprotonated carboxylic acid group (Figure 5a), showing behavior similar to that of GLY. Although there is no clear oxidation peak, this shoulder evidently increased with increasing concentration of AMPA, a trend also observed in the Ar-treated samples. The calculated Δ*E* values are similar to those of GLY, except for Pyd and Pyr, for which a higher affinity is observed.

Sarc is another compound that can be generated during the GLY degradation via the direct C–P lyase pathway.^62^ Being neutral at pH 7, it has the highest affinity for the CNW surfaces, as shown by the highest *K*_f_. With its amino group, it can interact with different carbon–oxygen (C–O) functional groups, mainly through hydrogen bonding and nucleophilic interactions. For all three analytes, the presence of hydrogen termination was beneficial to the detection according to the simulation. Indeed, it is interesting to note a positive correlation, for both AMPA and GLY, between the adsorption intensity and the area of the B3 Raman band (Figure 7d), which is related to the C–H stretching, while it is independent of Sarc.^42^

## Conclusions

This study explored the modification of B, N-codoped carbon nanowalls through various defect engineering strategies, primarily focusing on plasma treatment and boron incorporation, and their subsequent impact on the electrochemical oxidation of glyphosate, aminomethylphosphonic acid, and sarcosine. The Ar-plasma treatment predominantly induced chemical modifications, particularly in the formation and substitution of functional groups, leaving the BCNW morphology largely unaffected. A rapid initial process was observed, where part of the sp^2^-C and C–O bonds were removed, creating carbon vacancies, and resulting in defect sites and dangling bonds. These defects over time recombined with oxygen, giving rise to C=O functionalities. As the plasma treatment progressed, a transformation of these dangling bonds and defects into sp^3^-C configurations was noted, with Pyd and graphitic nitrogen functionalities increasing, while B–N functionalities decreased and Pyr nitrogen disappeared. The introduction of a diborane admixture not only influenced the boron doping content, but also significantly affected the distribution of C- and N-functional groups. A decrease in both sp^2^-C and sp^3^-C was observed with increasing boron concentration due to effective heteroatom substitution. The presence of diborane in the plasma also influenced the incorporation of nitrogen-containing functional groups, enhancing their reactivity, as evidenced by the BCNW morphology and OES measurements. Direct oxidation of GLY, AMPA, and Sarc was achieved. The effectiveness varied based on the boron-to-carbon (B/C) ratio and post-growth Ar plasma treatment durations. Lower B/C ratios (below 1k) resulted in higher electrical resistivity and larger ferro-ferricyanide peak-to-peak separations, making them less effective for analyte oxidation. The sample grown at a B/C ratio of 2000 ppm emerged as the most effective, enabling the oxidation of all analytes; interestingly, it possessed the lowest EASA, suggesting that, in this case, the surface area plays a secondary role compared to the nature of the defects. The affinity of AMPA and Sarc to BCNW slightly diminished with the prolonged Ar-plasma treatment, while GLY’s affinity saw a substantial decrease even after minimal plasma exposure. Nitrogen defects, particularly Pyd sites, played a pivotal role in GLY oxidation, inhibiting GLY adsorption due to charge repulsions, while we found B–C and B–N bonds to be preferential sites. Finally, along with the traditional characteristic ratios of carbon materials, we identified the D+D* peak at ≈2570 cm^–1^ to be inversely correlated with the B defects. In addition, molecular simulations predicted the strongest adsorption of GLY and AMPA on the hydrogen-terminated and hydroxyl defect sites on the modeled carbon structure, with interaction energies of −2.51 eV and comparable values. In contrast, the weakest interactions of 0.93 eV were found for pyridinic-nitrogen defects for GLY adsorption, suggesting either weak binding or possibly even molecular repulsion. Notably, pristine graphitic regions exhibited GLY adsorption energies similar to those of graphitic-nitrogen defects, implying analogous interaction modes. Overall, the highly favorable adsorption to hydrogen and hydroxyl defects is consistent with the fact that GLY and AMPA contain multiple hydrogen bonding moieties and hydrophilic groups. These computational results provide fundamental insights into the binding configurations, which can guide further experimental work on interfacing biological molecules with defective engineered carbon.

## Materials and Methods

GLY, AMPA (both PESTANAL, analytical standard), and Sarc (purity 98%) were purchased from Sigma-Aldrich. Potassium ferricyanide (K_3_[Fe(CN)_6_]) and potassium ferrocyanide trihydrate (K_4_[Fe(CN)_6_]%3H_2_O) were purchased from POCH (Poland). A stock phosphate solution (PBS, 0.1 M) was prepared by combining 8.7331 g of K_2_HPO_4_ and 125 μL of 85% H_3_PO_4_ in a 500 mL volumetric flask. All solutions were prepared with deionized water. Scanning electron microscopy (SEM) images were acquired utilizing an FEI Quanta 250 FEG (ThermoFisher Scientific) tool, fitted with a Schottky field emission gun, operating on secondary electrons at a 20 kV accelerating voltage. Scanning transmission electron microscopy (STEM) and electron energy loss spectroscopy (EELS) investigations were performed with probe Cs-corrected Titan^3^ G2 60-300 (ThermoFisher Scientific) microscope at the accelerating voltage of 300 kV. Bright field (BF) and high angle annular dark field (HAADF) techniques were used for imaging in STEM mode. X-ray photoelectron spectroscopy (XPS) examinations were conducted using an Escalab 250Xi (ThermoFisher Scientific) multispectroscope, operating with an AlKα X-ray source and a spot diameter of 650 μm. A pass energy of 20 eV was maintained through the hemispherical analyzer. Charge compensation was facilitated by a flow of low-energy electrons and Ar^+^ ions, with a final peak calibration at adventitious C 1s at 284.6 eV. The Raman spectra were measured using a LabRam Aramis Raman spectrometer from Horiba Jobin Yvon, equipped with an Olympus BX41 confocal microscope and a Synapse CCD camera from Horiba Jobin Yvon, with an integration time of 5s (20 averages) and diffraction grating of 300 gr/mm. Excitations were performed with 632.8 nm and 533 nm lasers. The optical emission spectra (OES) were collected by a UV–vis spectrometer (Ocean Optics), in the wavelength range of 300–900 nm, 3–5 mm above the sample surface, using a collimator and an optical fiber, with an integration time of 5 s; 20 individual acquisitions were then averaged.

### BCNW Synthesis and Modification

The BCNW electrodes were fabricated by a microwave plasma enhanced chemical vapor deposition (MPECVD) system (SEKI Technotron AX5400S, Japan) on 10 × 10 mm *p*-type silicon wafer slides <100>. Before deposition, the substrates underwent RCA cleaning, followed by ultrasonic treatment in acetone and isopropanol, rinsing, and seeding through ultrasonication in a water-based diamond slurry. The specific procedure can be found in our earlier research.^38,63^ The microwave power, process duration, and total pressure remained constant throughout. The temperature of the CVD stage was maintained at 700 °C, while the microwave power was set at 1300 W. Precursors such as H_2_, CH_4_, N_2_, and B_2_H_6_ were utilized for the synthesis of BCNW electrodes. A plasma cleaner system (Diener ZEPTO, Germany) was used to perform the plasma postgrowth treatment of the BCNW. At a pressure of 0.3 mbar, plasma RF with a frequency of 13.56 MHz, and a power of 300 W was employed for argon plasma exposure.

### EC Characterization and Analytes Oxidation

The electrochemical performance of the BCNW electrodes was evaluated by cyclic voltammetry (CV) and electrochemical impedance spectroscopy (EIS) using a VMP-300 BioLogic galvanostat/potentiostat from France, controlled by EC-lab software. All electrochemical studies were performed in a three-electrode cell system. The working electrode (WE) was a BCNW electrode with a surface area of 0.05 cm^2^, while the counter electrode (CE) and reference electrode (RE) were a platinum wire and Ag wire coated with AgCl, respectively. The electrolyte used for these investigations was a 2.5 mM [Fe(CN)_6_]^3–/4–^ (1:1) solution with 0.05 mol dm^–3^ phosphate buffer solution (PBS) as the supporting electrolyte. The cyclic voltammetry (CV) scan rate ranged from 5 to 500 mV s^–1^. Differential pulse voltammetry (DPV) was employed under the following conditions: the potential range was 0.2 to 1.6 V (vs Ag/AgCl) with a pulse width of 150 ms, a pulse height of 80 mV, a step width of 1000 ms, and a scan rate of 10 mV s^–1^. Electrochemical impedance spectroscopy (EIS) measurements were recorded at formal potential over a frequency range of 0.1 Hz to 100 kHz, with a peak-to-peak amplitude of 10 mV. The interface can be modeled by an equivalent electrical circuit (EEC) that includes the ohmic resistance of the electrolyte (*R*_s_), the Warburg impedance (*Z*_w_), the charge transfer resistance (*R*_ct_), and the constant phase element (CPE). The electrochemical double-layer capacitance (termed *C*_eff_) was determined using the following equation:
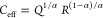
1

In this equation, α represents an additional parameter related to the constant phase element and falls within the range of 0 < α ≤ 1. The impedance (*Z*) of the CPE: *Z* = *Q*_0_ – 1(*i*ω)^−α^, where ω represents the angular frequency and *Q*_0_ is the CPE parameter in F s ^(α-1)^.

To fit the DPV results, a Freundlich-like adsorption isotherm was employed, since GLY is known to better fit such a model when adsorbed on carbon-based materials.^64^ The equation used is the following:
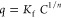
2where the Freundlich constant (*K*_f_) is related to the adsorption capacity of the electrode for the analyte; higher *K*_f_ values indicate a more pronounced current response in the DPV measurement, while 1/*n* indicates the heterogeneity of the adsorption surface and the intensity of adsorption, where for *n* > 1, it suggests cooperative adsorption, while, for *n* < 1, it becomes more difficult as the surface coverage increases.

### Quantum Modeling of System Surface

The atomistic modeling of BCNW surfaces with defects and targeted molecules (AMPA, GLY, and Sarc) was conducted using the QuantumATK v2023.09 software package from Synopsys. Moreover, the side configurations were expanded for structural defects, such as graphitic-boron, graphitic-nitrogen, pyridinic-nitrogen, and pyrrolic-nitrogen. For all calculations, the Universal Force Field UFF potential of Rappe, Casewit, Colwell, Goddard, and Skiff was utilized, with TremoloXcalculator.^65^ To study the interactions of the above molecules with the nanowall surface, these molecules were placed at fixed initial positions. The slabs were configured with periodic boundary conditions. The vacuum distance (attn. in the *z*-axis) between CNW surfaces was set to 14 Å to prevent additional interactions between graphene sublayers. The minimal energy delta for the convergence calculation was set to 0.05 eV/A, and the pressure was set to 0.01 GPa. Structures were relaxed using the limited memory Broyden–Fletcher–Goldfarb–Shanno method (L-BFGS).^66^ Moreover, slabs were submitted to charge equilibration.^67^ The optimal density of defects was calculated by finding the minimal energy configuration for every case and the total energy of the systems after system relaxation.

## Supporting Information Available

BCNW morphology (SEM, TEM, length), deconvoluted Raman spectroscopy results, XPS, electrochemical characterization (EIS and CV), material stability (DPV), and calculated adsorption energies by MD simulation (PDF)

## References

[ref1] Sobaszek M.; Siuzdak K.; Ryl J.; Sawczak M.; Gupta S.; Carrizosa S. B.; Ficek M.; Dec B.; Darowicki K.; Bogdanowicz R. Diamond Phase (Sp3-C) Rich Boron-Doped Carbon Nanowalls (Sp2-C): Physicochemical and Electrochemical Properties. J. Phys. Chem. C 2017, 121 (38), 20821–20833. 10.1021/acs.jpcc.7b06365.

[ref2] Sun T.; Zhang G.; Xu D.; Lian X.; Li H.; Chen W.; Su C. Defect Chemistry in 2D Materials for Electrocatalysis. Mater. Today Energy 2019, 12, 215–238. 10.1016/j.mtener.2019.01.004.

[ref3] Garcia-Segura S.; Vieira dos Santos E.; Martínez-Huitle C. A. Role of Sp3/Sp2 Ratio on the Electrocatalytic Properties of Boron-Doped Diamond Electrodes: A Mini Review. Electrochem. Commun. 2015, 59, 52–55. 10.1016/j.elecom.2015.07.002.

[ref4] Pierpaoli M.; Szopińska M.; Wilk B. K.; Sobaszek M.; Łuczkiewicz A.; Bogdanowicz R.; Fudala-Książek S. Electrochemical Oxidation of PFOA and PFOS in Landfill Leachates at Low and Highly Boron-Doped Diamond Electrodes. J. Hazard. Mater. 2021, 403, 123606 10.1016/j.jhazmat.2020.123606. 33264854

[ref5] Chen L.; Wang Y.; Cheng S.; Zhao X.; Zhang J.; Ao Z.; Zhao C.; Li B.; Wang S.; Wang S.; Sun H. Nitrogen Defects/Boron Dopants Engineered Tubular Carbon Nitride for Efficient Tetracycline Hydrochloride Photodegradation and Hydrogen Evolution. Appl. Catal. B Environ. 2022, 303, 120932 10.1016/j.apcatb.2021.120932.

[ref6] Yang L.; Jiang S.; Zhao Y.; Zhu L.; Chen S.; Wang X.; Wu Q.; Ma J.; Ma Y.; Hu Z. Boron-doped carbon nanotubes as metal-free electrocatalysts for the oxygen reduction reaction. Angew. Chem. 2011, 123 (31), 7270–7273. 10.1002/ange.201101287. 21688363

[ref7] Liu Z.; Zhao Z.; Wang Y.; Dou S.; Yan D.; Liu D.; Xia Z.; Wang S. In Situ Exfoliated, Edge-Rich, Oxygen-Functionalized Graphene from Carbon Fibers for Oxygen Electrocatalysis. Adv. Mater. 2017, 29 (18), 1606207 10.1002/adma.201606207. 28276154

[ref8] Kim S.; Ievlev A. V.; Jakowski J.; Vlassiouk I. V.; Sang X.; Brown C.; Dyck O.; Unocic R. R.; Kalinin S. V.; Belianinov A.; et al. Multi-Purposed Ar Gas Cluster Ion Beam Processing for Graphene Engineering. Carbon 2018, 131, 142–148. 10.1016/j.carbon.2018.01.098.

[ref9] Shen A.; Zou Y.; Wang Q.; Dryfe R. A. W.; Huang X.; Dou S.; Dai L.; Wang S. Oxygen Reduction Reaction in a Droplet on Graphite: Direct Evidence That the Edge Is More Active than the Basal Plane. Angew. Chem. 2014, 126 (40), 10980–10984. 10.1002/ange.201406695. 25124986

[ref10] Zhang C.; Huang N.; Zhai Z.; Liu L.; Chen B.; Yang B.; Jiang X.; Yang N. Bifunctional Oxygen Electrocatalyst of Co4N and Nitrogen-Doped Carbon Nanowalls/Diamond for High-Performance Flexible Zinc–Air Batteries. Adv. Energy Mater. 2023, 13 (41), 2301749 10.1002/aenm.202301749.

[ref11] Tang C.; Wang H. F.; Chen X.; Li B. Q.; Hou T. Z.; Zhang B.; Zhang Q.; Titirici M. M.; Wei F. Topological Defects in Metal-Free Nanocarbon for Oxygen Electrocatalysis. Adv. Mater. 2016, 28 (32), 6845–6851. 10.1002/adma.201601406. 27167616

[ref12] Rukhlyada K. A.; Matytcina V. V.; Baldina A. A.; Volkova O.; Kozodaev D. A.; Barakova N. V.; Orlova O. Y.; Smirnov E.; Skorb E. V. Universal Method Based on Layer-by-Layer Assembly for Aptamer-Based Sensors for Small-Molecule Detection. Langmuir 2023, 39 (31), 10820–10827. 10.1021/acs.langmuir.3c00822. 37490765

[ref13] Lach P.; Garcia-Cruz A.; Canfarotta F.; Groves A.; Kalecki J.; Korol D.; Borowicz P.; Nikiforow K.; Cieplak M.; Kutner W.; et al. Electroactive Molecularly Imprinted Polymer Nanoparticles for Selective Glyphosate Determination. Biosens. Bioelectron. 2023, 236, 115381 10.1016/j.bios.2023.115381. 37267687

[ref14] Bro̷nstad J. O.; Friestad H. O. Method for Determination of Glyphosate Residues in Natural Waters Based on Polarography of the N-Nitroso Derivative. Analyst 1976, 101 (1207), 820–824. 10.1039/an9760100820. 984423

[ref15] Cao Y.; Wang L.; Shen C.; Wang C.; Hu X.; Wang G. An Electrochemical Sensor on the Hierarchically Porous Cu-BTC MOF Platform for Glyphosate Determination. Sensors Actuators, B Chem. 2019, 283, 487–494. 10.1016/j.snb.2018.12.064.

[ref16] Pintado S.; Montoya M. R.; Rodríguez-Amaro R.; Mayén M.; Mellado J. M. R. Electrochemical Determination of Glyphosate in Waters Using Electrogenerated Copper Ions. Int. J. Electrochem. Sci. 2012, 7 (3), 2523–2530. 10.1016/S1452-3981(23)13898-3.

[ref17] Moraes F. C.; Mascaro L. H.; Machado S. A. S.; Brett C. M. A. Direct Electrochemical Determination of Glyphosate at Copper Phthalocyanine/Multiwalled Carbon Nanotube Film Electrodes. Electroanalysis 2010, 22 (14), 1586–1591. 10.1002/elan.200900614.

[ref18] Dubbin W. E.; Sposito G.; Zavarin M. X-Ray Absorption Spectroscopic Study of Cu-Glyphosate Adsorbed by Microcrystalline Gibbsite. Soil Sci. 2000, 165 (9), 699–707. 10.1097/00010694-200009000-00003.

[ref19] Undabeytia T.; Morillo E.; Maqueda C. FTIR Study of Glyphosate–Copper Complexes. J. Agric. Food Chem. 2002, 50 (7), 1918–1921. 10.1021/jf010988w. 11902933

[ref20] Oliveira P. C. P. A.; Maximiano E. M.; Oliveira P. C. P. A.; Camargo J. S.; Fiorucci A. R.; Arruda G. J. Direct Electrochemical Detection of Glyphosate at Carbon Paste Electrode and Its Determination in Samples of Milk, Orange Juice, and Agricultural Formulation. J. Environ. Sci. Heal. - Part B Pestic. Food Contam. Agric. Wastes 2018, 53 (12), 817–823. 10.1080/03601234.2018.1505081. 30325268

[ref21] Caceres-Jensen L.; Rodríguez-Becerra J.; Sierra-Rosales P.; Escudey M.; Valdebenito J.; Neira-Albornoz A.; Dominguez-Vera V.; Villagra C. A. Electrochemical Method to Study the Environmental Behavior of Glyphosate on Volcanic Soils: Proposal of Adsorption-Desorption and Transport Mechanisms.. J. Hazard. Mater. 2019, 379, 120746 10.1016/j.jhazmat.2019.120746. 31276919

[ref22] Pierpaoli M.; Dettlaff A.; Szopińska M.; Karpienko K.; Wróbel M.; Łuczkiewicz A.; Fudala-Książek S.; Bogdanowicz R. Simultaneous Opto-Electrochemical Monitoring of Carbamazepine and Its Electro-Oxidation by-Products in Wastewater. J. Hazard. Mater. 2021, 419, 126509 10.1016/j.jhazmat.2021.126509. 34323723

[ref23] Pierpaoli M.; Lewkowicz A.; Dec B.; Nadolska M.; Bogdanowicz R. Impedimetric Sensing of α-Amino Acids Driven by Micro-Patterned 1,8-Diazafluoren-9-One into Titania- Boron- Doped Maze-like Nanocarbons. Sensors Actuators B Chem. 2022, 371, 132459 10.1016/j.snb.2022.132459.

[ref24] Pierpaoli M.; Szopińska M.; Olejnik A.; Ryl J.; Fudala-Ksiażek S.; Łuczkiewicz A.; Bogdanowicz R. Engineering Boron and Nitrogen Codoped Carbon Nanoarchitectures to Tailor Molecularly Imprinted Polymers for PFOS Determination. J. Hazard. Mater. 2023, 458, 131873 10.1016/j.jhazmat.2023.131873. 37379604

[ref25] Cervantes-Sodi F.; Csányi G.; Piscanec S.; Ferrari A. C. Edge-Functionalized and Substitutionally Doped Graphene Nanoribbons: Electronic and Spin Properties. Phys. Rev. B 2008, 77 (16), 165427 10.1103/PhysRevB.77.165427.

[ref26] Pierpaoli M.; Ficek M.; Jakobczyk P.; Karczewski J.; Bogdanowicz R. Self-Assembly of Vertically Oriented Graphene Nanostructures: Multivariate Characterisation by Minkowski Functionals and Fractal Geometry. Acta Mater. 2021, 214, 116989 10.1016/j.actamat.2021.116989.

[ref27] Lajaunie L.; Pardanaud C.; Martin C.; Puech P.; Hu C.; Biggs M. J.; Arenal R. Advanced Spectroscopic Analyses on a: C-H Materials: Revisiting the EELS Characterization and Its Coupling with Multi-Wavelength Raman Spectroscopy. Carbon 2017, 112, 149–161. 10.1016/j.carbon.2016.10.092.

[ref28] Ewels P.; Sikora T.; Serin V.; Ewels C. P.; Lajaunie L. A Complete Overhaul of the Electron Energy-Loss Spectroscopy and X-Ray Absorption Spectroscopy Database: Eelsdb.Eu. Microsc. Microanal. 2016, 22 (3), 717–724. 10.1017/S1431927616000179. 26899024

[ref29] Zhai Z.; Zhang C.; Chen B.; Xiong Y.; Liang Y.; Liu L.; Yang B.; Yang N.; Jiang X.; Huang N. Covalently-Bonded Diaphite Nanoplatelet with Engineered Electronic Properties of Diamond. Adv. Funct. Mater. 2024, 2401949 (9), 1–11. 10.1002/adfm.202401949.

[ref30] Ghosh S.; Ganesan K.; Polaki S. R.; Mathews T.; Dhara S.; Kamruddin M.; Tyagi A. K. Influence of Substrate on Nucleation and Growth of Vertical Graphene Nanosheets. Appl. Surf. Sci. 2015, 349, 576–581. 10.1016/j.apsusc.2015.05.038.

[ref31] Ferrari A. C.; Robertson J. Origin of the 1150 – Cm–1 Raman Mode in Nanocrystalline Diamond. Phys. Rev. B 2001, 63 (12), 2–5. 10.1103/PhysRevB.63.121405.

[ref32] Kuzmany H.; Pfeiffer R.; Salk N.; Günther B. The Mystery of the 1140 Cm-1 Raman Line in Nanocrystalline Diamond Films. Carbon 2004, 42 (5–6), 911–917. 10.1016/j.carbon.2003.12.045.

[ref33] Teii K.; Ikeda T.; Fukutomi A.; Uchino K. Effect of Hydrogen Plasma Exposure on the Amount of Trans-Polyacetylene in Nanocrystalline Diamond Films. J. Vac. Sci. Technol. B Microelectron. Nanom. Struct. 2006, 24 (1), 263 10.1116/1.2163885.

[ref34] Lee A. Y.; Yang K.; Anh N. D.; Park C.; Lee S. M.; Lee T. G.; Jeong M. S. Raman Study of D* Band in Graphene Oxide and Its Correlation with Reduction. Appl. Surf. Sci. 2021, 536, 147990 10.1016/j.apsusc.2020.147990.

[ref35] Wang J. J.; Zhu M. Y.; Outlaw R. A.; Zhao X.; Manos D. M.; Holloway B. C.; Mammana V. P. Free-Standing Subnanometer Graphite Sheets. Appl. Phys. Lett. 2004, 85 (7), 1265–1267. 10.1063/1.1782253.

[ref36] Sankaran K. J.; Ficek M.; Kunuku S.; Panda K.; Yeh C.-J.-J.; Park J. Y.; Sawczak M.; Michałowski P. P.; Leou K.-C.-C.; Bogdanowicz R.; et al. Self-Organized Multi-Layered Graphene-Boron-Doped Diamond Hybrid Nanowalls for High-Performance Electron Emission Devices. Nanoscale 2018, 10 (3), 1345–1355. 10.1039/C7NR06774G. 29296984

[ref37] Lazar P.; Mach R.; Otyepka M. Spectroscopic Fingerprints of Graphitic, Pyrrolic, Pyridinic, and Chemisorbed Nitrogen in N-Doped Graphene. J. Phys. Chem. C 2019, 123 (16), 10695–10702. 10.1021/acs.jpcc.9b02163.

[ref38] Pierpaoli M.; Ficek M.; Rycewicz M.; Sawczak M.; Karczewski J.; Ruello M. L.; Bogdanowicz R. Tailoring Electro/Optical Properties of Transparent Boron-Doped Carbon Nanowalls Grown on Quartz. Materials 2019, 12 (3), 547 10.3390/ma12030547. 30759814 PMC6385157

[ref39] Wang G.; Li X.; Wang Y.; Zheng Z.; Dai Z.; Qi X.; Liu L.; Cheng Z.; Xu Z.; Tan P.; et al. Interlayer Coupling Behaviors of Boron Doped Multilayer Graphene. J. Phys. Chem. C 2017, 121 (46), 26034–26043. 10.1021/acs.jpcc.7b05771.

[ref40] Pardanaud C.; Martin C.; Roubin P.; Giacometti G.; Hopf C.; Schwarz-Selinger T.; Jacob W. Raman Spectroscopy Investigation of the H Content of Heated Hard Amorphous Carbon Layers. Diam. Relat. Mater. 2013, 34, 100–104. 10.1016/j.diamond.2013.02.009.

[ref41] Pardanaud C.; Cartry G.; Lajaunie L.; Arenal R.; Buijnsters J. G. Investigating the Possible Origin of Raman Bands in Defective Sp2/Sp3 Carbons below 900 Cm–1: Phonon Density of States or Double Resonance Mechanism at Play?. C J. Carbon Res. 2019, 5 (4), 79 10.3390/c5040079.

[ref42] Casiraghi C.; Ferrari A. C.; Robertson J. Raman Spectroscopy of Hydrogenated Amorphous Carbons. Phys. Rev. B 2005, 72 (8), 1–14. 10.1103/PhysRevB.72.085401.

[ref43] Kurita S.; Yoshimura A.; Kawamoto H.; Uchida T.; Kojima K.; Tachibana M.; Molina-Morales P.; Nakai H. Raman Spectra of Carbon Nanowalls Grown by Plasma-Enhanced Chemical Vapor Deposition. J. Appl. Phys. 2005, 97 (10), 104320 10.1063/1.1900297.

[ref44] Ma Z.; Tsounis C.; Kumar P. V.; Han Z.; Wong R. J.; Toe C. Y.; Zhou S.; Bedford N. M.; Thomsen L.; Ng Y. H.; et al. Enhanced Electrochemical CO2 Reduction of Cu@CuxO Nanoparticles Decorated on 3D Vertical Graphene with Intrinsic Sp3-Type Defect. Adv. Funct. Mater. 2020, 30 (24), 1–12. 10.1002/adfm.201910118.

[ref45] Li D.; Ren B.; Jin Q.; Cui H.; Wang C. N.-D. Oxygen-Functionalized, Edge- and Defect-Rich Vertically Aligned Graphene for Highly Enhanced Oxygen Evolution Reaction. J. Mater. Chem. A 2018, 6 (5), 2176–2183. 10.1039/c7ta07896j.

[ref46] Liu Y.; Li M.; Zhou B.; Xuan X.; Li H. Flexible BN Co-Doped Graphene Electrodes for Electrochemical Detection of Serotonin in Bodily Fluids. Electrochim. Acta 2023, 457, 142494 10.1016/j.electacta.2023.142494.

[ref47] Figueras M.; Villar-Garcia I. J.; Vines F.; Sousa C.; Illas F. Correcting Flaws in the Assignment of Nitrogen Chemical Environments in N-Doped Graphene. J. Phys. Chem. C 2019, 123 (17), 11319–11327. 10.1021/acs.jpcc.9b02554.

[ref48] Dhillon S.; Kant R. Theory for Electrochemical Impedance Spectroscopy of Heterogeneous Electrode with Distributed Capacitance and Charge Transfer Resistance. J. Chem. Sci. 2017, 129 (8), 1277–1292. 10.1007/s12039-017-1335-x.

[ref49] Friedl J.; Stimming U. Determining Electron Transfer Kinetics at Porous Electrodes. Electrochim. Acta 2017, 227, 235–245. 10.1016/j.electacta.2017.01.010.

[ref50] Chung T. Y.; Tsao C. S.; Tseng H. P.; Chen C. H.; Yu M. S. Effects of Oxygen Functional Groups on the Enhancement of the Hydrogen Spillover of Pd-Doped Activated Carbon. J. Colloid Interface Sci. 2015, 441, 98–105. 10.1016/j.jcis.2014.10.062. 25490569

[ref51] Bi H.; Li Y.; Liu S.; Guo P.; Wei Z.; Lv C.; Zhang J.; Zhao X. S. Carbon-Nanotube-Modified Glassy Carbon Electrode for Simultaneous Determination of Dopamine, Ascorbic Acid and Uric Acid: The Effect of Functional Groups. Sensors Actuators, B Chem. 2012, 171–172, 1132–1140. 10.1016/j.snb.2012.06.044.

[ref52] Guo X.; Hou Y.; Chen X.; Zhang R.; Li W.; Tao X.; Huang Y. Tuning the Structural Stability and Electrochemical Properties in Graphene Anode Materials by B Doping: A First-Principles Study. Phys. Chem. Chem. Phys. 2022, 24 (35), 21452–21460. 10.1039/D2CP02730E. 36048145

[ref53] Zhan C.; Zhang Y.; Cummings P. T.; Jiang D. E. Enhancing Graphene Capacitance by Nitrogen: Effects of Doping Configuration and Concentration. Phys. Chem. Chem. Phys. 2016, 18 (6), 4668–4674. 10.1039/C5CP06952A. 26794824

[ref54] Pierpaoli M.; Jakóbczyk P.; Dec B.; Giosuè C.; Czerwińska N.; Lewkowicz A.; Ruello M. L.; Bogdanowicz R. A Novel Hierarchically-Porous Diamondized Polyacrylonitrile Sponge-like Electrodes for Acetaminophen Electrochemical Detection. Electrochim. Acta 2022, 430, 141083 10.1016/j.electacta.2022.141083.

[ref55] Niedziałkowski P.; Cebula Z.; Malinowska N.; Białobrzeska W.; Sobaszek M.; Ficek M.; Bogdanowicz R.; Anand J. S.; Ossowski T. Comparison of the Paracetamol Electrochemical Determination Using Boron-Doped Diamond Electrode and Boron-Doped Carbon Nanowalls. Biosens. Bioelectron. 2019, 126, 308–314. 10.1016/j.bios.2018.10.063. 30445306

[ref56] Esmaeilian A.; Dionysiou D. D.; O’Shea K. E. Incorporating Simultaneous Effect of Initial Concentration and Sorbent Dose into Removal Prediction Model Using Glyphosate Experimental Data and Theoretical Analysis. Chem. Eng. J. 2022, 445, 136667 10.1016/j.cej.2022.136667.

[ref57] Santos J. S.; Pontes M. S.; Santiago E. F.; Fiorucci A. R.; Arruda G. J. An Efficient and Simple Method Using a Graphite Oxide Electrochemical Sensor for the Determination of Glyphosate in Environmental Samples. Sci. Total Environ. 2020, 749, 142385 10.1016/j.scitotenv.2020.142385. 33370922

[ref58] Ayiania M.; Smith M.; Hensley A. J. R.; Scudiero L.; McEwen J. S.; Garcia-Perez M. Deconvoluting the XPS Spectra for Nitrogen-Doped Chars: An Analysis from First Principles. Carbon 2020, 162, 528–544. 10.1016/j.carbon.2020.02.065.

[ref59] Li O. L.; Prabakar K.; Kaneko A.; Park H.; Ishizaki T. Exploration of Lewis Basicity and Oxygen Reduction Reaction Activity in Plasma-Tailored Nitrogen-Doped Carbon Electrocatalysts. Catal. Today 2019, 337, 102–109. 10.1016/j.cattod.2019.02.058.

[ref60] Li B.; Sun X. Y.; Su D. Calibration of the Basic Strength of the Nitrogen Groups on the Nanostructured Carbon Materials. Phys. Chem. Chem. Phys. 2015, 17 (10), 6691–6694. 10.1039/C4CP05765A. 25673108

[ref61] Lan H.; Jiao Z.; Zhao X.; He W.; Wang A.; Liu H.; Liu R.; Qu J. Removal of glyphosate from water by electrochemically assisted MnO_2_ oxidation process. Sep. Purif. Technol. 2013, 117, 30–34. 10.1016/j.seppur.2013.04.012.

[ref62] Marangoni D. G.; Smith R. S.; Roscoe S. G. Surface Electrochemistry of the Oxidation of Glycine at Pt. Can. J. Chem. 1989, 67 (5), 921–926. 10.1139/v89-141.

[ref63] de Freitas Araújo K. C.; Vieira dos Santos E.; Pierpaoli M.; Ficek M.; Santos J. E. L.; Martínez-Huitle C. A.; Bogdanowicz R. Diamondized Carbon Nanoarchitectures as Electrocatalytic Material for Sulfate-Based Oxidizing Species Electrogeneration. Electrochim. Acta 2022, 430, 141069 10.1016/j.electacta.2022.141069.

[ref64] Herath G. A. D.; Poh L. S.; Ng W. J. Statistical Optimization of Glyphosate Adsorption by Biochar and Activated Carbon with Response Surface Methodology. Chemosphere 2019, 227, 533–540. 10.1016/j.chemosphere.2019.04.078. 31004820

[ref65] Schneider J.; Hamaekers J.; Chill S. T.; Smidstrup S.; Bulin J.; Thesen R.; Blom A.; Stokbro K. ATK-ForceField: A New Generation Molecular Dynamics Software Package. Model. Simul. Mater. Sci. Eng. 2017, 25 (8), 085007 10.1088/1361-651X/aa8ff0.

[ref66] Liu D. C.; Nocedal J. On the Limited Memory BFGS Method for Large Scale Optimization. Math. Program 1989, 45 (1–3), 503–528. 10.1007/BF01589116.

[ref67] Rappe A. K.; Goddard W. A. Charge Equilibration for Molecular Dynamics Simulations. J. Phys. Chem. 1991, 95 (8), 3358–3363. 10.1021/j100161a070.

