# Supplementary material for: Tailoring Defects in B, N-Codoped Carbon Nanowalls for Direct Electrochemical Oxidation of Glyphosate and its Metabolites
Source: ACS Appl Mater Interfaces. 2024 Jul 5;16(28):36784–95. doi: 10.1021/acsami.4c04478 (PMC11261608; doi:10.1021/acsami.4c04478)
Supplement: Supplementary file 1 — am4c04478_si_001.pdf [file am4c04478_si_001.pdf]

Supporting Information

Tailoring Defects in N, B-Codoped Carbon Nanowalls for Direct Electrochemical Oxidation of Glyphosate and its Metabolites

Mattia Pierpaoli[a*], Pawel Jakobczyk[a], Mateusz Ficek[a], Bartłomiej Dec[a], Jacek Ryl[b], Bogdan Rutkowski[c], Aneta Lewkowicz[d], Robert Bogdanowicz[a]

[a] Faculty of Electronics, Telecommunications and Informatics, Gdańsk University of Technology, 11/12 Gabriela Narutowicza Street, 80-233 Gdańsk, Poland

[b] Institute of Nanotechnology and Materials Engineering, Gdańsk University of Technology, 11/12 Gabriela Narutowicza Street, 80-233 Gdańsk, Poland

[c] AGH University of Krakow, Faculty of Metals Engineering and Industrial Computer Science, al. A. Mickiewicza 30, 30-059 Krakow, Poland;

[d] Faculty of Mathematics, Physics and Informatics, University of Gdańsk, Wita Stwosza 57, 80-308 Gdańsk, Poland

[*] Corresponding authors: *mattia.pierpaoli@pg.edu.pl* [+48 (58) 347 15 03]

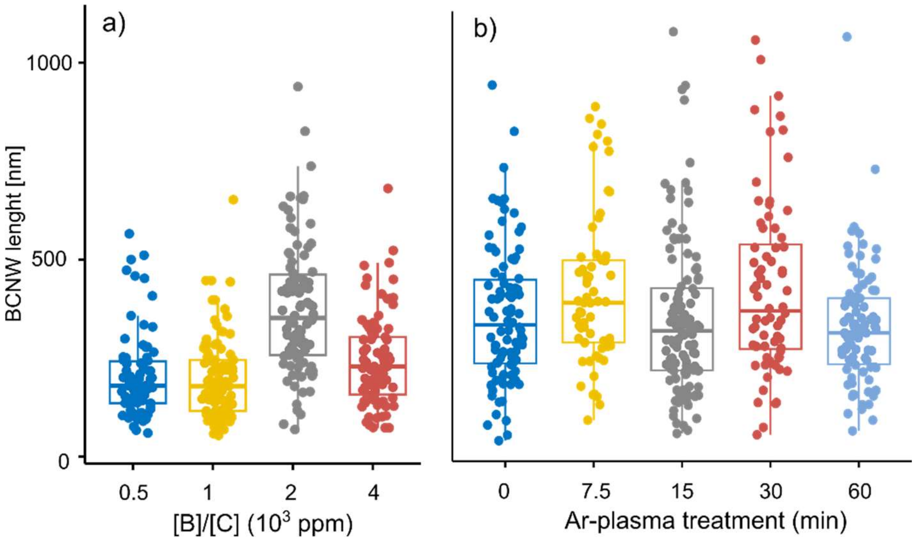

Figure S1 – Distribution of the BCNW length as a function of (a) the [B]/[C] and (b) Ar-plasma treatment.

Table S1 –Significance test for differences between different BCNW groups

group1	group2	p	p.adj	p.format	p.signif	method
0.5	1	5.77e- 1	5.8 e- 1	0.5773	ns	Wilcoxon
0.5	2	5.88E-14	2.90E-13	5.90E-14	****	Wilcoxon
0.5	4	1.02e- 2	2 e- 2	0.0102	*	Wilcoxon
1	2	1.22E-15	7.30E-15	1.20E-15	****	Wilcoxon
1	4	2.82e- 3	8.5 e- 3	0.0028	**	Wilcoxon
2	4	8.21e- 9	3.30e- 8	8.20E-09	****	Wilcoxon

group1	group2	p	p.adj	p.format	p.signif	method
0	7.5	0.0784	0.39	0.0784	ns	Wilcoxon
0	15	0.397	1	0.3975	ns	Wilcoxon
0	30	0.0603	0.36	0.0603	ns	Wilcoxon
0	60	0.288	1	0.2883	ns	Wilcoxon
7.5	15	0.00977	0.078	0.0098	**	Wilcoxon
7.5	30	1	1	1	ns	Wilcoxon
7.5	60	0.00481	0.048	0.0048	**	Wilcoxon
15	30	0.0112	0.079	0.0112	*	Wilcoxon
15	60	0.956	1	0.9558	ns	Wilcoxon
30	60	0.00568	0.051	0.0057	**	Wilcoxon

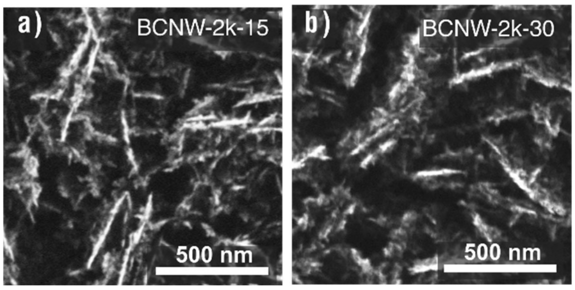

Figure S2 - SEM images of the BCNW-2k after Ar-plasma treatment at different durations.

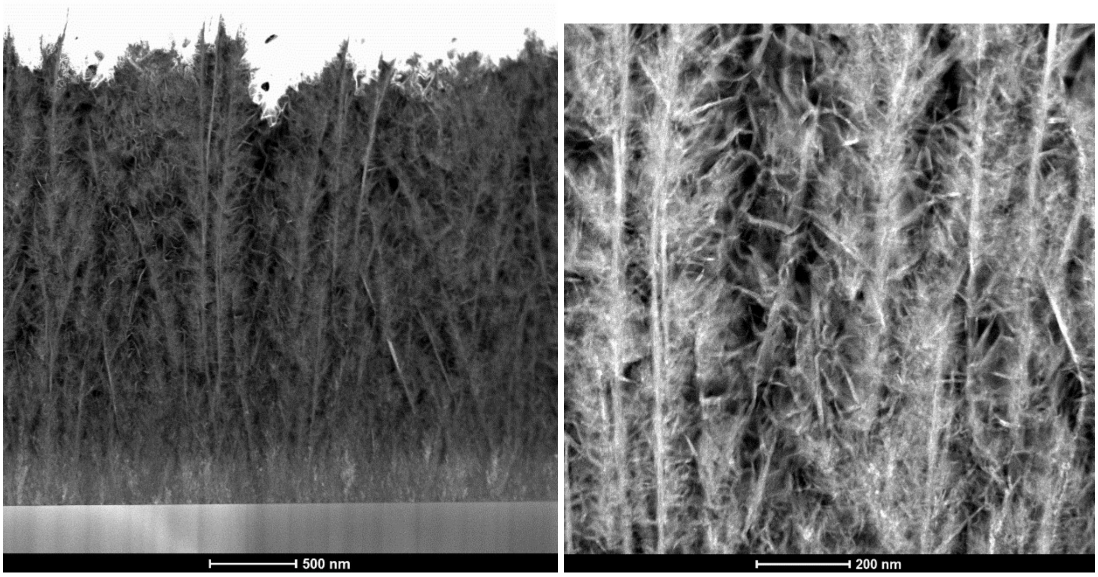

Figure S3 - Overview of the sample (a) and BNCW in detail (b), STEM-HAADF

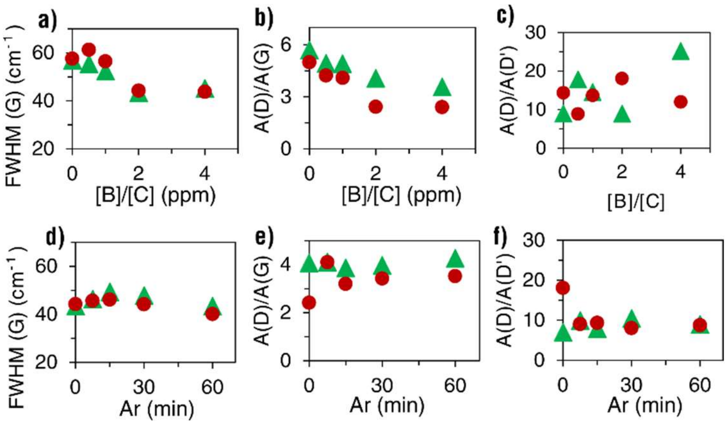

Figure S4 – Characteristic Raman values and ratios derived from the fitted results using a 533nm laser (green triangle) and 632.8nm laser (red circle)

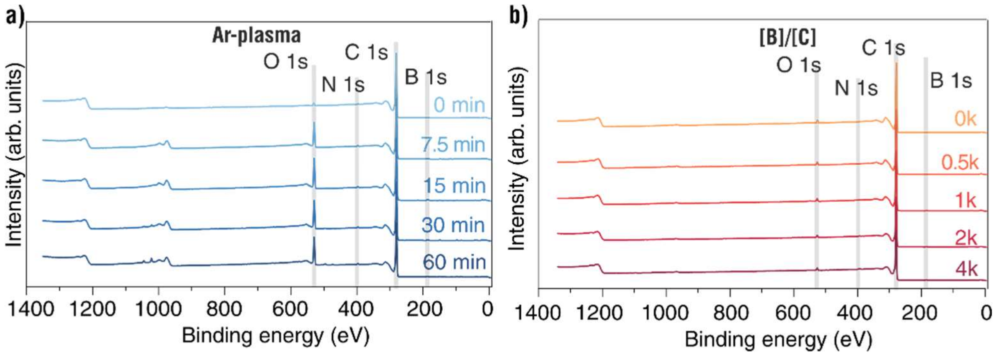

Figure S5 – XPS survey of (a) Ar-treated and different (b) [B]/[C] ratio samples

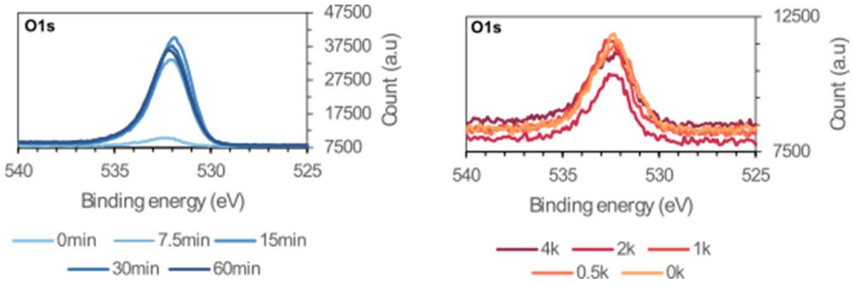

Figure S6- (a) High-resolution XPS spectra of the oxygen region (O1s) at different Ar-treatment durations and [B]/[C]

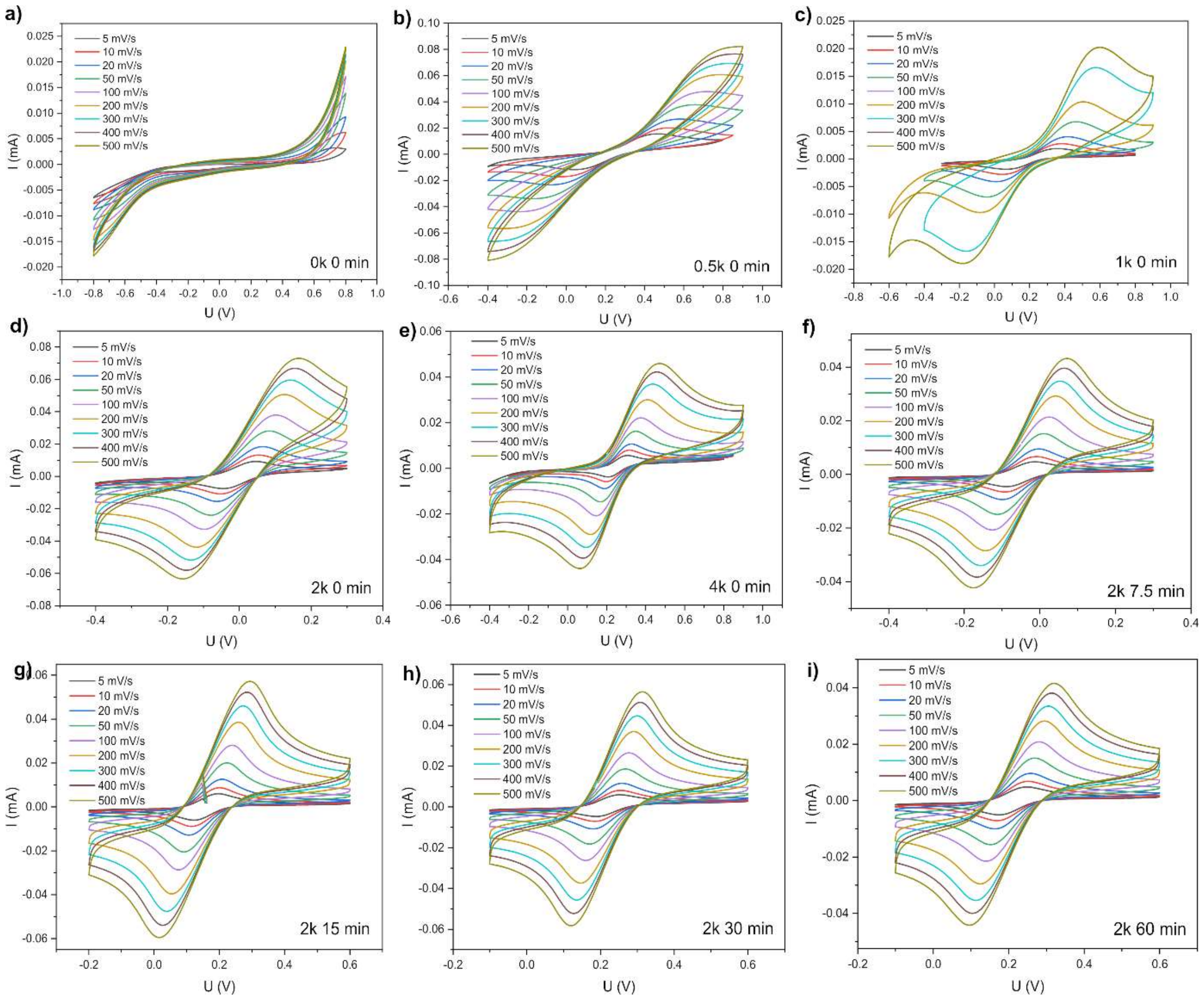

Figure S7 – Cyclic voltammograms at different scan rates for different BCNW samples

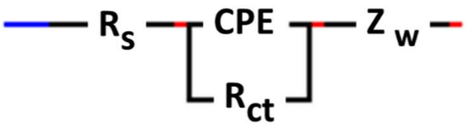

Figure S8 – EEC used to fit the EIS data

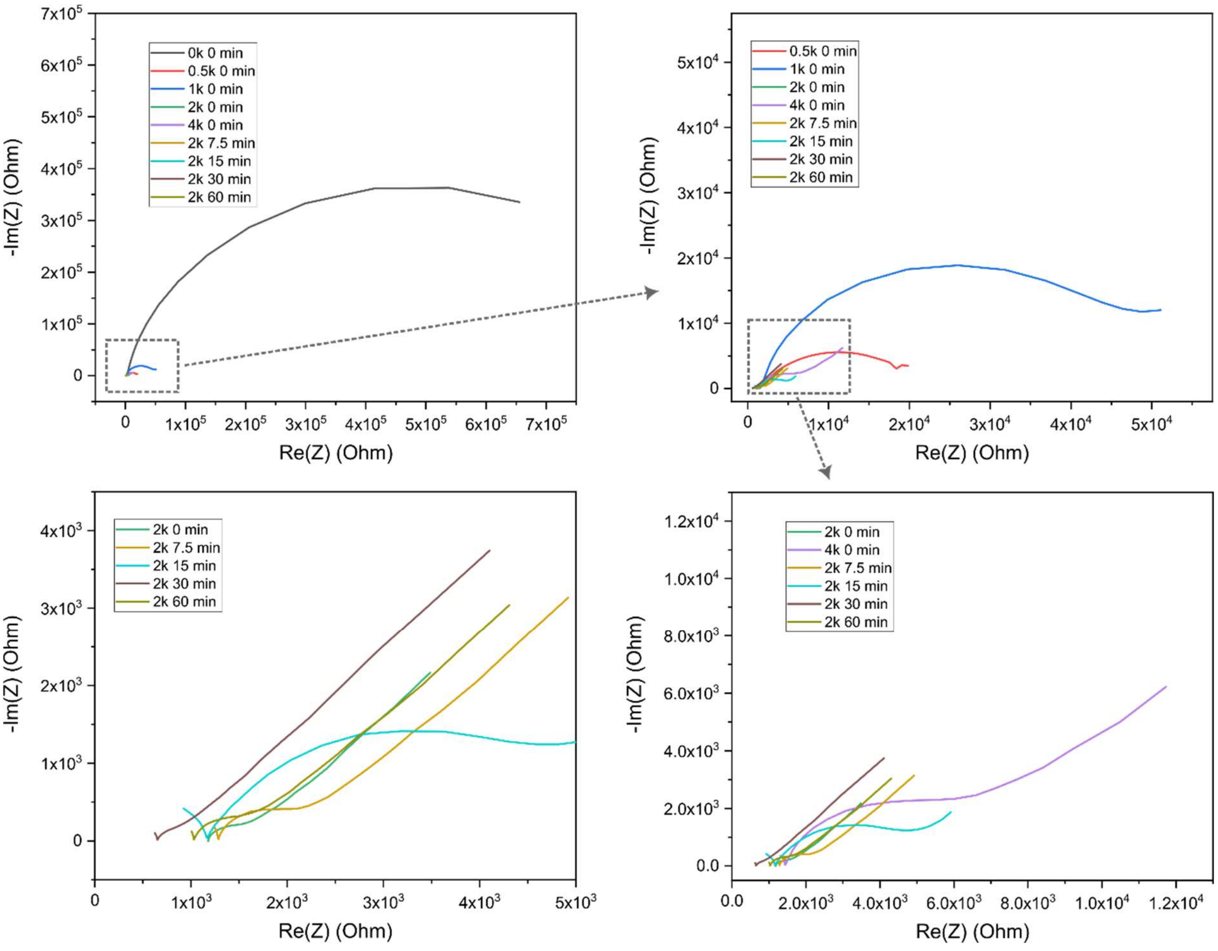

Figure S9 – EIS results of the different samples

Table S2 - Slopes obtained from dependence of peak current of cyclic voltammetry of 2.5 mM $[Fe(CN)_6]^{3-/4-}$ in 0.05 M PBS on the square root of scan rates, measured using BCNW electrodes with different level of doping and after different time of argon plasma treatment.

**BCNW**	**Slopes/ (mA·(mV·s^{-1})$^{-1/2}$)**	
	Anodic	Cathodic
0k	-	-
0.5k	0.0051	-0.00470
1k	0.001	0.00095
2k	0.00345	-0.00298
4k	0.00213	-0.00200
2k_7.5 min	0.002	-0.00196
2k_15 min	0.00265	-0.00275
2k_30 min	0.00257	-0.00263
2k_60 min	0.00194	-0.00204

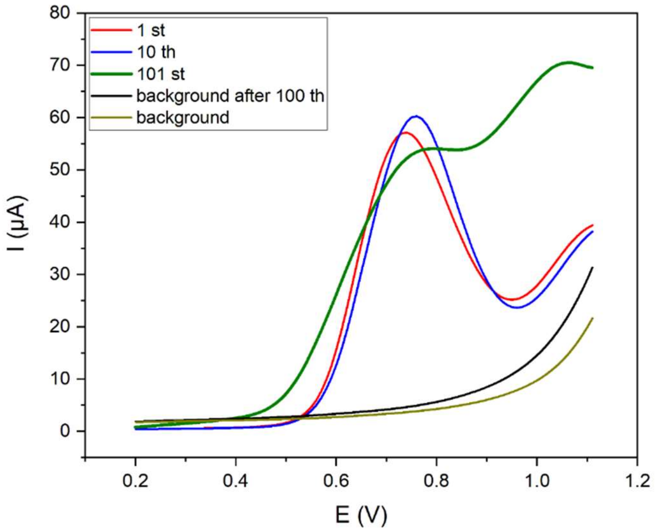

Figure S10 – DPV of glyphosate after 1, 10 and 101 oxidation cycles, with background for comparison.

Table S3 – Calculated adsorption energies ΔE of studied analytes at different BCNW defects.

	Vacuum				Water			
	deGly	AMPA	deAMPA	Sarcosine	deGly	AMPA	deAMPA	Sarcosine
O	13.41	14.18	13.48	14.39	-2.51	-1.07	-0.87	0.06
Pyd	15.51	16.1	15.41	16.47	0.93	1.58	-0.05	0.21
g-N	15.7	16.29	15.58	16.63	-1.1	-0.63	-0.14	1.53
Pyr	-0.46	0.05	-0.41	0.68	-0.45	-0.71	-2.13	0.49
g-B	-0.1	0.3	-0.36	0.8	0.6	2.14	0.16	1.87
